# Oncolytic virus driven T-cell-based combination immunotherapy platform for colorectal cancer

**DOI:** 10.3389/fimmu.2022.1029269

**Published:** 2022-11-03

**Authors:** Mathieu J. F. Crupi, Zaid Taha, Thijs J. A. Janssen, Julia Petryk, Stephen Boulton, Nouf Alluqmani, Anna Jirovec, Omar Kassas, Sarwat T. Khan, Sydney Vallati, Emily Lee, Ben Zhen Huang, Michael Huh, Larissa Pikor, Xiaohong He, Ricardo Marius, Bradley Austin, Jessie Duong, Adrian Pelin, Serge Neault, Taha Azad, Caroline J. Breitbach, David F. Stojdl, Michael F. Burgess, Scott McComb, Rebecca Auer, Jean-Simon Diallo, Carolina S. Ilkow, John Cameron Bell

**Affiliations:** ^1^ Centre for Innovative Cancer Research, Ottawa Hospital Research Institute, Ottawa, ON, Canada; ^2^ Department of Biochemistry, Microbiology and Immunology, University of Ottawa, Ottawa, ON, Canada; ^3^ Discovery Research, Turnstone Biologics Inc, Ottawa, ON, Canada; ^4^ Human Health Therapeutics Research Centre, National Research Council, Ottawa, ON, Canada

**Keywords:** oncolytic virus, T cell engager, CEA, FAP, CTLA4

## Abstract

Colorectal cancer is the third most diagnosed cancer and the second leading cause of cancer mortality worldwide, highlighting an urgent need for new therapeutic options and combination strategies for patients. The orchestration of potent T cell responses against human cancers is necessary for effective antitumour immunity. However, regression of a limited number of cancers has been induced by immune checkpoint inhibitors, T cell engagers (TCEs) and/or oncolytic viruses. Although one TCE has been FDA-approved for the treatment of hematological malignancies, many challenges exist for the treatment of solid cancers. Here, we show that TCEs targeting CEACAM5 and CD3 stimulate robust activation of CD4 and CD8-positive T cells in *in vitro* co-culture models with colorectal cancer cells, but *in vivo* efficacy is hindered by a lack of TCE retention in the tumour microenvironment and short TCE half-life, as demonstrated by HiBiT bioluminescent TCE-tagging technology. To overcome these limitations, we engineered Bispecific Engager Viruses, or BEVirs, a novel tumour-targeted vaccinia virus platform for intra-tumour delivery of these immunomodulatory molecules. We characterized virus-mediated TCE-secretion, TCE specificity and functionality from infected colorectal cancer cells and patient tumour samples, as well as TCE cytotoxicity in spheroid models, in the presence and absence of T cells. Importantly, we show regression of colorectal tumours in both syngeneic and xenograft mouse models. Our data suggest that a different profile of cytokines may contribute to the pro-inflammatory and immune effects driven by T cells in the tumour microenvironment to provide long-lasting immunity and abscopal effects. We establish combination regimens with immune checkpoint inhibitors for aggressive colorectal peritoneal metastases. We also observe a significant reduction in lung metastases of colorectal tumours through intravenous delivery of our oncolytic virus driven T-cell based combination immunotherapy to target colorectal tumours and FAP-positive stromal cells or CTLA4-positive T_reg_ cells in the tumour microenvironment. In summary, we devised a novel combination strategy for the treatment of colorectal cancers using oncolytic vaccinia virus to enhance immune-payload delivery and boost T cell responses within tumours.

## Introduction

Colorectal cancer (CRC) is the third most common cancer diagnosed worldwide and the second most lethal cancer in both men and women due to its early-onset, late-stage diagnosis and therapeutic resistance (1). For patients with metastatic CRC, the prognosis remains poor with 5-year survival rates below 20% due to the limited effectiveness of standard of care chemotherapy or targeted therapies (2). Over the last decade, immunotherapy has begun to drastically alter treatment paradigms for some cancers, including CRC where PD-1 blockade (3, 4) produced durable responses in patients with high levels of microsatellite instability and mismatch repair deficiency (MSI-H/dMMR) tumours. Immunotherapeutic agents such as oncolytic viruses (5, 6), bispecific T cell engagers (TCEs) (7, 8), chimeric antigen receptor T-cell therapy (CAR T) (9), and immune checkpoint inhibitors targeting programmed death 1 (PD-1) or cytotoxic T-lymphocyte associated protein 4 (CTLA4) (10) function primarily by inducing/triggering T cell-mediated antitumour responses and overcoming barriers present in the hostile tumour microenvironment (TME). Despite recent successes, the lack of response to current immunotherapies in non-MSI-H/dMMR CRC (11), which constitute the majority of metastatic CRC cases (12), highlight the critical need for additional immunotherapeutic approaches.

The TME is composed of heterogeneous populations of tumour cells, cancer-associated fibroblasts (CAFs), and other cell types that interact together to support tumour growth and spread, but also to promote or maintain immune inhibition. Analyses of the interaction network between tumour cells and their microenvironment have elucidated some of the putative mechanisms of immunotherapy resistance (13, 14). Single-cell transcriptome studies (15, 16) have demonstrated that in CRC, the TME shifts towards a suppressive cellular immunity marked by an enrichment of regulatory T (T_reg_) cells, stromal myofibroblasts/CAFs, and loss or downregulation of HLA on cancer cells. Combination immunotherapeutic strategies that attack several attributes of a hostile TME can lower the immune setpoint threshold (17) and enhance the overall survival of patients with metastatic CRC. TCEs are immunomodulatory molecules consisting of two linked single-chain variable fragments (scFv) that target a tumour associated antigen and CD3 on T cells. By binding to CD3, TCEs can force T cells to interact with tumour cells regardless of mutational status, HLA expression or co-stimulation. Upon recruitment of T cells by TCEs, pseudo-immunological synapse formation triggers the release of perforin and granzyme resulting in tumour-specific cell lysis. The first FDA-approved TCE known as Blinatumomab has been used for the treatment of hematological malignancies (8). Other TCEs have been developed to target different cell-surface proteins including carcinoembryonic antigen-related cell adhesion molecule 5 (CEA) (18, 19), which is highly expressed in CRC and other cancers and is known to drive metastasis (20, 21). CEA mutants may also inhibit TGFβ signalling, which alters the microbiome to promote CRC (22). However, a TCE therapy has not been successful in clinical trials using intravenous (IV) administration to target CEA-positive CRC (18, 19). Low therapeutic efficacy may be explained by exclusion of infiltrating immune cells from the TME, the short half-life of TCEs which prevents penetration into solid tumours, or subtherapeutic TCE doses due to treatment-related toxicities (7).

One strategy to overcome the limitations of TCE therapy includes the use of oncolytic viruses as a transgene delivery mechanism (23). Oncolytic viruses are naturally occurring or genetically modified to replicate within and kill cancer cells, but also provide the benefit of recruiting immune cells into the TME (5). Viral susceptibility is determined by cancer-specific defects in signalling pathways, including cellular growth control and innate antiviral defence systems (24). We and others have hypothesized that using oncolytic viruses as a delivery mechanism for TCEs will result in tumour-localized expression of TCEs and improved therapeutic outcomes of solid tumours (24–33). This approach allows for high local TCE delivery, reduced off-target toxicity compared to systemic TCE administration, and generates a bystander killing effect in the TME by directly engaging T cells to kill uninfected cancer cells. In combination with release of tumour antigens caused by viral oncolysis, this strategy enables *in situ* tumour vaccination and continued antitumour immunity (5). Here, we engineer Bispecific Engager Viruses (BEVirs), as a tumour-targeted oncolytic virus platform for *in situ* tumour delivery of TCE immunomodulatory molecules. We selected a modified oncolytic Copenhagen strain of vaccinia virus (VV; ClinicalTrials.gov: NCT04301011 and patent publication 20220056480) as a therapeutic vector based on its ability to infect and kill human and murine CRC cell lines as spheroid models more efficiently than other oncolytic viruses. VV also has many desirable features which include, but are not limited to: [A] replication within the TME - using mouse models and patient-derived tumour explants, we have shown that VV has an inherent ability to attack aspects of the TME including the tumour neo-vasculature (34) and certain CAF populations (35); [B] the capacity to encode large and multiple therapeutic transgenes (5); [C] extensive clinical knowledge of the viral vector safety due to its use as a vaccine to eradicate smallpox (5) and treat other diseases (36, 37), and [D] oncolytic VV can be selectively delivered to CRC tumours by IV administration (38) and clinical trials have demonstrated safety in CRC patients (38, 39). Systemic delivery of therapeutics is of particularly importance for CRC treatment as many patients present with locally advanced or metastatic disease.

Importantly, we characterize the ability of infected cells to secrete TCEs targeting CEA, as well as TCE specificity and functionality from infected colorectal cancer cells, patient tumour samples, and spheroid models in the presence and absence of T cells. Treatment of colorectal tumours with BEVirs reduced tumour regression and increased survival of both syngeneic and xenograft mouse models. Our data indicate that a unique profile of cytokines induced by replication of BEVirs within the tumour may contribute to the pro-inflammatory and immune effects driven by T cells in the TME to provide long-lasting immunity and abscopal effects. As it is now evident that combination therapies are necessary to cure aggressive tumour models (40–47), we establish BEVir combination regimens with T_reg_-targeting αCTLA4 (48, 49), alone or expressed by virus, for aggressive colorectal peritoneal metastases. We also observe a significant reduction in lung metastases of colorectal tumours through intravenous delivery of our BEVir targeting CEA on tumour cells, in combination with oncolytic VV encoding a TCE targeting FAP on CAFs or αCTLA4. Thus, our data show that combination viro-immunotherapy can drive T-cell responses in tumours as an impactful strategy for the challenging treatment of aggressive CRCs.

## Results

### Generation and validation of TCEs targeting CRC cells

**Figure 1:**
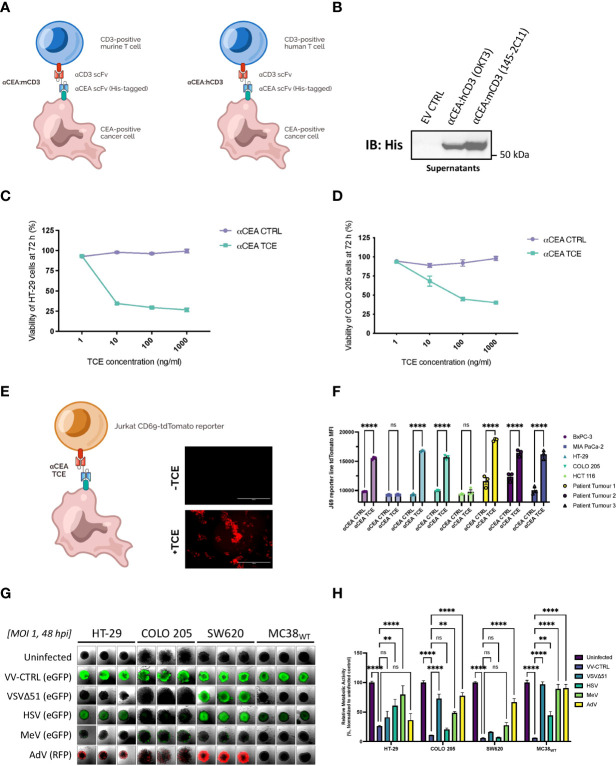
Generation and validation of TCEs targeting CRC cells to encode into oncolytic vaccinia virus as an optimal delivery system. **(A)** Two novel TCEs were designed by linking a scFv that binds human CEA on the surface of cancer cells to a scFv that recognizes CD3ε on either murine T cells (αCEA:mCD3) or human T cells (αCEA:hCD3) which can be CD4 or CD8 positive. **(B)** HEK293T cells were transiently transfected with αCEA TCE constructs or pcDNA3.1 empty vector control (EV) and incubated for 48 h. TCE-containing supernatants were collected, spun down to remove cell debris, and concentrated using centrifugal filter units with a 10 kDa molecular weight cutoff. Samples were quantified by BCA assay to load 10 µg of supernatant per lane, separated by SDS-PAGE, and immunoblots were probed with a His antibody to detect His-tagged TCEs. No His tag was detected for the EV control as expected. **(C)** HT-29 cells were co-cultured with human PBMCs (E:T = 5:1) and αCEA TCE (αCEA:hCD3) or αCEA CTRL (αCEA:mCD3) at indicated concentrations. Resazurin assay was performed at 72 h to determine cancer cell viability after TCE treatment. Results show relative % ± SEM. **(D)** COLO 205 cells were co-cultured with human PBMCs (E:T = 5:1) and αCEA TCE (αCEA:hCD3) or αCEA CTRL (αCEA:mCD3) at indicated concentrations. Resazurin assay was performed at 72 h to determine cancer cell viability after TCE treatment. Results show relative % ± SEM. **(E)** In co-cultures with HT-29 cells that express CEA and Jurkat CD69-tdTomato reporter cells (J69; T cells were modified by CRISPR to express tdTomato under the control of the CD69 promoter), the addition of TCEs (1 µg) leads to the visualization of tdTomato-positive Jurkat J69 cells (E:T = 1:1). Scale bar = 400 µm. **(F)** Three patient-derived CEA-positive colorectal cancer cell lines and other CEA-positive cells (BxPC-3, HT-29, COLO 205) activate J69 cells in co-cultures with αCEA TCE (αCEA:hCD3), but not with αCEA CTRL (αCEA:mCD3) or CEA-negative control cell lines (MIA PaCa-2, HCT 116). Results show MFI ± standard error of the mean (SEM); Two-way ANOVA with Sidak’s correction for multiple comparisons. **(G)** HT-29, COLO 205, SW620 and MC38_WT_ spheroids were infected at an MOI 1 of oncolytic vaccinia virus (Copenhagen/Cop strain) VV-CTRL, oncolytic Vesicular Stomatitis virus (VSVΔ51), oncolytic Herpes simplex virus (HSV), oncolytic Measles virus (MeV), or oncolytic Adenovirus (AdV). Spheroids were imaged at 48 hpi to detect transgene expression of enhanced green fluorescent protein (eGFP) or red fluorescent protein (RFP). All colorectal cancer spheroids expressed abundant eGFP levels after VV-CTRL infection, compared to other viruses. **(H)** Spheroid viability was assessed in triplicate by resazurin assay at 120 hpi, relative to uninfected controls. VV-CTRL decreased cell viability of HT-29, COLO205, SW620 and MC38_WT_ spheroids. vaccinia virus was able to infect all the colorectal cancer cell lines as spheroids. VV-CTRL decreased cell viability of HT-29, COLO205, SW620 and MC38_WT_ spheroids. Other viruses also decreased spheroid viability to a lesser extent. Of note, MeV did not significantly change the viability of MC38_WT_ spheroids, as expected, since this oncolytic virus do not infect murine cancer cell lines but served as a control. Results show relative % ± SEM; Two-way ANOVA.

We designed two novel TCEs by linking a scFv that recognizes CD3ε on either murine T cells (αCEA:mCD3) or human T cells (αCEA:hCD3) to a His-tagged scFv that binds human CEACAM5 on the surface of cancer cells (**Figure 1A**; **Supplementary Figure 1A**). The high affinity scFv targeting CEA was derived from MFE-23, a monoclonal antibody used in patients with colorectal cancer tumours (50); whereas the scFv that binds murine or human CD3ε was derived from well-characterized OKT3 or 145-2C11 antibodies (51), respectively (**Figure 1A**; **Supplementary Figure 1A**). Herein, the TCE that recognizes the appropriate, species-specific CD3ε in our experiments is referred to as αCEA TCE, and the TCE that serves as a negative control for CD3ε binding (opposite species) is referred to as αCEA CTRL (**Figure 1B**). An upstream Ig Kappa leader sequence was added upstream of the TCE to promote secretion from cells. TCE expression and secretion from transiently transfected HEK293T cells were confirmed by immunoblotting using an anti-His tag antibody (**Figure 1B**). We then developed a pipeline to collect, purify and concentrate TCEs from supernatants of transfected cells, which we use to validate TCE constructs in downstream cell-based and *in vivo* experiments (**Supplementary Figure 1B**). To confirm that our TCEs interacted specifically with CEA on the surface of cancer cells, we developed a binding assay using an Alexa Fluor 647-conjugated His antibody to quantify TCE attachment to cell-surface CEA on cancer cells (**Supplementary Figure 1C**). We showed that the αCEA TCE specifically binds to CEA-positive CRC cells (HT-29, COLO 205; murine MC38 cells expressing human CEA/MC38_CEA_), pancreatic cancer cells (BxPC-3), breast cancer cells (MCF7) and lung cancer cells (A549), but not CEA-negative cells including glioblastoma U87MG and MC38_WT_ (**Supplementary Figure 1D**). These results were consistent with CEA levels in different cancer cell lines (**Supplementary Figure 1E**).

Functionality and cytotoxicity of the αCEA TCE were quantified using *in vitro* co-culture models with human CRC cell lines that express CEA, including HT-29 (**Figure 1C**) and COLO 205 (**Figure 1D**), and human PBMCs (Effector : Target Cell = 5:1). Cell viability of CRC cells decreased, in a dose-dependent manner, in the presence of αCEA TCE, but not αCEA CTRL (**Figures 1C, D**). Additionally, there was no significant change in cell viability of CEA-negative HCT 116 CRC cells upon co-culture with PBMCs and αCEA TCE (**Supplementary Figure 2A**). We also showed that increasing amounts of αCEA TCE, but not αCEA CTRL, promote a greater reduction in cell viability of pancreatic cancer cell lines that express CEA (BxPC-3; **Supplementary Figure 2B**), without altering cell viability of CEA-negative cells (MIA PaCa-2; **Supplementary Figure 2C**). These data demonstrate that our engineered TCEs specifically recognize cell-surface CEA regardless of tumour type. In addition, αCEA CTRL did not cross-react with the opposite species of CD3ε T cell population, as no effect was observed with this negative control in co-cultures.

### TCE therapy leads to T cell activation with CRC cell lines and patient tumour samples

We next investigated the extent of TCE-mediated T cell activation against target CRC cell lines in co-culture experiments. Using CD69 as a marker of T cell activation, we measure activation of both CD4-positive and CD8-positive T cell populations in PBMCs co-cultured with HT-29 cells in the absence and presence of αCEA TCE or αCEA CTRL by flow cytometry (**Supplementary Figure 2D**). When co-cultured with HT-29 cells and the αCEA TCE, there was a 70-80% increase in the percentage of CD4 and CD8 T cells expressing CD69 in comparison to PBMCs treated in the absence of HT-29 cells or treated with the αCEA CTRL. We also measured T cell activation against a collection of CEA-positive or CEA-negative cell lines, including three patient-derived CEA-positive colorectal cancer cell lines, using a Jurkat reporter cell line (J69), modified by CRISPR to express tdTomato under the control of the CD69 promoter. In co-cultures with cancer cell lines expressing CEA as well as the three patient-derived CEA-positive colorectal cancer cell lines, the addition of TCEs leads to the visualization of tdTomato-positive Jurkat cells (**Figures 1E, F**). In contrast, cell lines, such as MIA PaCa-2 and HCT-116, which do not express CEA, led to low tdTomato expression, and all cell lines treated with the αCEA CTRL TCE resulted in negligible tdTomato signal. Altogether, the results demonstrate that the αCEA TCE can specifically activate T cells against CEA-positive cell lines, including heterogeneous tumour samples from CRC patients.

To determine whether chemotherapy can sensitize to TCE therapy, we treated cells with the current standard of care drug for CRC, 5-Fluorouracil (5-FU), which can enhance CEA levels (52). We showed that different doses of 5-FU increase CEA levels in COLO 205 and SW620 CRC cell lines that normally express low levels of the protein (**Supplementary Figure 2E**). We did not detect any increase in CEA expression in HCT 116 and HCT15 CRC cell lines that lack CEA at basal levels, and we did not detect a noticeable increase in HT-29 cells that express abundant basal levels of CEA (**Supplementary Figure 2E**). These data suggest that 5-FU may synergize with TCE therapy as the drug can upregulate CEA expression on CRC cells with low levels of expression, sensitizing cells to TCE binding. Indeed, we showed that 5-FU enhances αCEA TCE binding to the surface of COLO 205 and SW620 cell lines (**Supplementary Figure 2F**), consistent with the antigen level increase (**Supplementary Figure 2E**). This TCE binding assay suggests that 5-FU may sensitize a heterogenous colorectal tumour in a patient to TCE therapy targeting CEA.

### Oncolytic vaccinia virus as an optimal delivery system for TCEs to target colorectal cancer

As a novel strategy to track our TCE *in vivo*, we are the first to our knowledge to generate TCEs fused to a HiBiT peptide for bioluminescent quantitation (**Supplementary Figure 3A**). We first examined the dynamic range of two HiBiT fusion constructs, where the 11-amino-acid sequence was added either to the αCD3 scFv (HiBiT_A_) or the αCEA scFv (HiBiT_B_) of the TCE (**Supplementary Figure 3A**). We transiently transfected the two individual constructs into HEK293T cells and collected lysates and supernatants at 48 h. Both TCE fusion constructs were detected in lysates by immunoblotting (**Supplementary Figure 3B**) and probing with Large BiT (LgBiT), the complementary polypeptide that binds HiBiT with high affinity. To verify the reconstitution and activation of the enzyme, in the presence of substrate, we measured the luminescent signal from the supernatant transfected cells (**Supplementary Figure 3C**). We detected higher levels of luminescence with HiBiT_B_ in a dynamic range that can be used to measure TCEs *in vivo*. These data suggest that HiBiT-tagging at the αCEA scFv (HiBiT_B_), in place of the original His tag, renders the TCE more accessible to substrate binding than HiBiT-tagging at the αCD3 scFv (HiBiT_A_), and our subsequent investigations warrant further use of the HiBiT_B_ construct. Using our established pipeline (**Supplementary Figure 1B**), we concentrated αCEA TCE-HiBiT_B_ to treat mice bearing colorectal tumours (MC38_WT_). We injected a single dose of 10 µg of TCE intratumourally and quantified the presence of a luminescent signal *ex vivo* from collected mouse serum or dissociated tumours at different time points after treatment. We show that the luminescent signal decreases over time in tumours (**Supplementary Figure 3D**), suggesting that the TCE has a short half-life, consist with other studies. In addition, we detect TCE presence in the serum which also decreases overtime (**Supplementary Figure 3D**), indicating that the tumour is leaky and may not retain all the TCE in the TME. Of note, even though others have shown that a HiBiT tag has low immunogenicity, we did not use this version of our TCE construct for subsequent *in vivo* studies as we did not want a potential confounder in our survival studies. These findings highlight some of the previously identified challenges of TCE delivery into solid tumours and support the rationale of improving delivery of these immunotherapeutics for CRC.

We analyzed a small library of oncolytic viral platforms to identify an ideal candidate that was broadly tropic towards human and murine colorectal cancer cell lines. We infected spheroids derived from HT-29, COLO 205, SW620 and MC38_WT_ cancer cell lines at an MOI 1 with either oncolytic VV (modified Copenhagen strain; VV-CTRL), oncolytic Herpes simplex virus (HSV), oncolytic Vesicular Stomatitis virus (VSVΔ51), oncolytic Measles virus (MeV), or oncolytic Adenovirus (AdV), all of which encode fluorescent proteins to observe the extent of infection. At 48 hours post-infection (hpi), spheroids were imaged to visualize the spread of enhanced green fluorescent protein (eGFP) or red fluorescent protein (RFP) transgenes expressed from the viruses (**Figure 1G**). VV-CTRL had the broadest tropism of all OVs tested with the capability of thoroughly infecting all four CRC spheroid models (**Figure 1G**). AdV and HSV infected only three of the models and did notably worse in the murine cell line, while VSV and MeV did poorly in most of the cell lines tested. We also assessed viability of the infected spheroids, relative to uninfected controls, at 120 hpi (**Figure 1H**). As expected, based on the fluorescent reporter expression, VV-CTRL also had the highest cytotoxicity of all the viruses in all cell lines tested. These results indicate that oncolytic vaccinia virus is an ideal platform for infection of colorectal tumours and delivery of a therapeutic transgene.

### Strategically combining oncolytic vaccinia virus and TCEs targeting CEA

**Figure 2:**
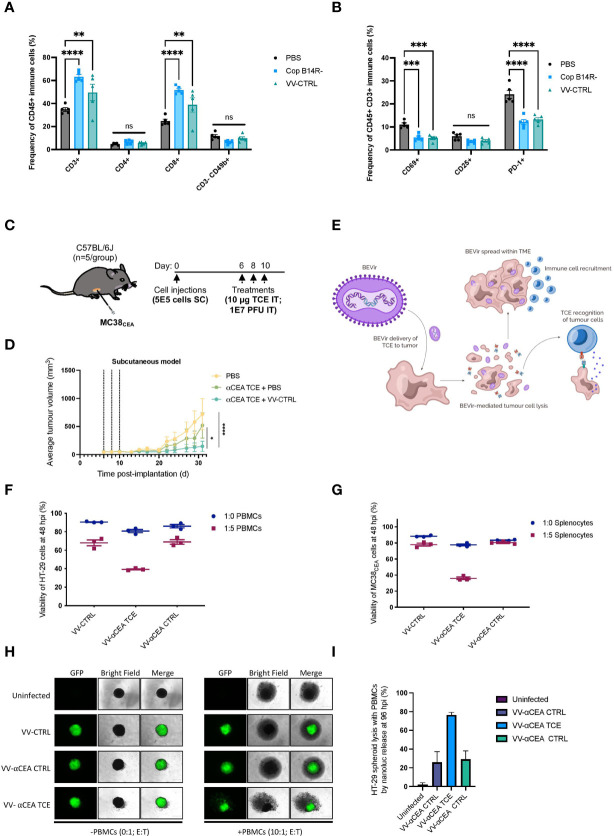
TCEs can activate T cells recruited to the tumour microenvironment by oncolytic vaccinia virus. **(A)** Two doses of vaccinia B14R-deleted (Cop B14R-) or oncolytic VV-CTRL, but not PBS control, both led to recruitment of CD8-positive T cells into MC38_WT_ tumours at 5 days post-intratumoural injections. In contrast, NK cells (CD3- CD49b+) were not recruited into tumours. Results show the frequency of CD45+ immune cells in % ± SEM, as determined by flow cytometry; Two-way ANOVA. **(B)** Virally recruited T cells did not express CD69 or CD25 activation markers, and T cells also did not express PD-1 as a marker of T cell exhaustion at this time post-infection. Results show the frequency of CD45+ CD3+ immune cells in % ± SEM, as determined by flow cytometry; Two-way ANOVA. **(C)** C57BL/6J mice bearing MC38_CEA_ subcutaneous tumours (implanted at 5E5 cells on day 0) were treated with 3 doses of 10 µg of αCEA TCE with or without 3 doses of VV-CTRL at 1E7 pfu at days 6, 8, and 10. **(D)** Average MC38_CEA_ tumour volumes overtime, showing improved efficacy in decreasing tumour burden with the combination treatment of VV-CTRL and αCEA TCE, compared to PBS control and αCEA TCE alone; Two-way ANOVA. **(E)** Schematic illustrating a Bispecific-Engager Virus (BEVir), a genetically modified oncolytic vaccinia strain (Cop 3p-5p-/VV-CTRL) encoding one of our validated TCEs targeting CEA and either human or murine T cells. Upon infection of CRC cell lines, the oncolytic vaccinia recruits T cells into the TME that can be activate by TCEs that recognize CEA on uninfected cells. T-cell mediated cell death can lead to a bystander killing effect. **(F)** Co-cultures with HT-29 cancer cells were infected 24 h after seeding at MOI 0.1. Inoculation medium was removed after 2 h and new medium was added containing human effector cells (E:T = 5:1, or 0:1 as a negative control). Cell viability was assessed by resazurin assay and decreased the most in the presence of human effector cells (PBMCs) and VV-αCEA TCE (αCEA:hCD3), compared to conditions with parental VV-CTRL or VV-αCEA CTRL (αCEA:mCD3). Results show relative % ± SEM. **(G)** Co-cultures with MC38_CEA_ cancer cells were infected 24 h after seeding at MOI 0.01. Inoculation medium was removed after 2 h and new medium was added containing murine effector cells (E:T = 5:1, or 0:1 as a negative control). Cell viability was assessed by resazurin assay and decreased the most in the presence of murine effector cells (splenocytes) and VV-αCEA TCE (αCEA:mCD3), compared to conditions with parental VV-CTRL or VV-αCEA CTRL (αCEA:hCD3). Results show relative % ± SEM. **(H)** HT-29-NLuc spheroids were grown in a methylcellulose matrix for 2 d before infecting with VV-CTRL, VV-αCEA TCE (αCEA:hCD3), or VV-αCEA CTRL (αCEA:mCD3). At 48 hpi, we added PBMCs (E:T = 10:1) or no PBMCs (E:T = 0:1) as a control, as indicated. At 96 hpi, spheroids were imaged, and we observed EGFP transgene expression. Scale bar = 500 µm. **(I)** NLuc release in media, as a surrogate measure for HT-29 cell death, was measured. A significant increase in luminescence was detected for VV-αCEA TCE with PBMCs added, compared to other virus controls and no PBMC conditions. Results show relative % ± SEM.

To investigate the immune-impact of oncolytic vaccinia virus on T cells in the colorectal cancer TME, we treated mice bearing subcutaneous MC38_WT_ tumours intratumourally with two doses at 1E7 pfu of vaccinia virus harbouring either a B14R deletion (Cop B14R-), or 5p, 3p and B14R deletions (VV-CTRL). We harvested tumours 5 days after the last virus injection and assessed the virus-mediated effects on T cells in the TME. Both viruses recruited CD8-positive T cells into the tumour (**Figure 2A**; **Supplementary Figure 4A**). Tumour infiltrating T cells did not express significant levels of T cell activation markers CD69 or CD25, or PD-1 (**Figure 2B**; **Supplementary Figure 4A**). These findings emphasize the benefit of oncolytic vaccinia virus as a platform to convert an immunologically ‘cold’ solid tumour into a ‘hot’ TME enriched with T cells for TCEs to be effective. Although the T cells are not activated, encoding TCEs into the virus may allow for enhanced T cell activation.

An initial screen was performed to assess the potential synergy of combining oncolytic vaccinia virus and a TCE targeting CEA. Upon confirmation that CEA depletion by siRNA did not negatively impact oncolytic vaccinia virus replication (**Supplementary Figure 5A**), we treated mice bearing murine MC38 cells expressing human CEA/MC38_CEA_ tumours with 3 doses of 10 μg of αCEA TCE with or without 3 doses of VV-CTRL at 1E7 pfu (**Figure 2C**). We measured average tumour volumes overtime and showed that tumour burden was significantly reduced with the combination treatment of VV-CTRL and αCEA TCE, compared to PBS and αCEA TCE combination or PBS alone control (**Figure 2D**). These data further suggest that encoding a TCE into oncolytic vaccinia virus could further increase the therapeutic efficacy of TCE therapy in preclinical CRC models bearing CEA-positive tumours.

### Engineering oncolytic vaccinia virus to encode TCEs targeting CEA

To generate a Bispecific Engager Virus (BEVir), we modified the genome of oncolytic vaccinia virus (VV-CTRL) to encode our validated TCEs targeting CEA and either human or murine T cells. We predict that vaccinia virus will recruit T cells into the tumour, but that inclusion of a secreted TCE will promote T cell activation and cancer cell death to create a bystander killing effect in the TME (**Figure 2E**). Plasmids with appropriate homology arms were designed and used to insert transgenes into the B14R/3p locus of the vaccinia virus genome by homologous recombination. Infection with oncolytic vaccinia virus (Cop 3p-5p-; no fluorescent tag) and transfection of the plasmid allowed for recombination to occur (**Supplementary Figure 5C**). Fluorescent plaques were selected, purified and sequenced to generate 3 viruses expressing: eGFP only (VV-CTRL); αCEA:mCD3 + eGFP + (VV-αCEA:mCD3), as well as αCEA:hCD3 + eGFP (VV-αCEA:hCD3). Herein, the oncolytic vaccinia virus that produces the TCE that recognizes the appropriate CD3ε is referred to as VV-αCEA TCE, and the oncolytic vaccinia virus that produces the TCE that serves as a negative control for CD3ε binding (opposite species) is referred to as VV-αCEA CTRL.

Visualization of eGFP upon infection of HEK293T cells (**Supplementary Figure 5D**) and secretion of TCEs from infected HEK293T cells was detected at different multiplicities of infection (MOIs) and at different time points by immunoblotting the collected supernatants for the His tag on the TCE (**Supplementary Figure 5E**). Similarly, we observed eGFP in HT-29 cells (**Supplementary Figure 6A**) and MC38_CEA_ cells (**Supplementary Figure 6B**) at 48 hpi. We detected the production of TCEs in both cell lysates and supernatants of HT-29 cells (**Supplementary Figure 6C**) and MC38_CEA_ cells (**Supplementary Figure 6D**). The insertion of a TCE transgene into VV did not interfere with viral replication (**Supplementary Figures 7A, C, E, G, I**) or viral lytic activity (**Supplementary Figures 7B, D, F, H, J**) in multiple human and murine cancer cell lines that possess or lack CEA expression. Thus, HT-29 and MC38_CEA_ cells could be used as ideal colorectal cancer models to study therapeutic outcomes in preclinical immunocompromised and immunocompetent mice, respectively.

To validate the activity of TCEs expressed from the viruses, human and murine tumour cell and effector co-culture experiments were performed. Co-cultures were prepared and at 24 h cancer cells were infected at multiple MOIs. Inoculation medium was removed after 2 h and new medium was added containing effector cells (E:T = 5:1, or 0:1 as a negative control). In the human co-culture model using PBMCs, HT-29 cell viability decreased the most in the presence of human effector cells and VV-αCEA:hCD3 (relevant αCEA TCE) compared to conditions with parental VV-CTRL or VV-αCEA:mCD3 (αCEA CTRL TCE) (**Figure 2F**). To better visualize cell populations, virus infection and cell death within co-cultures, HT-29 cells expressing the fluorescent marker Azurite were generated and infected with VV-αCEA:hCD3, which generates eGFP-positive plaques, and PBMCs stained with a red CellTracker dye (**Supplementary Figure 8A**). Lastly, we infected HEK293T cells with our VV-CTRL, VV-αCEA:hCD3, and VV-αCEA:mCD3 viruses, filtered out the vaccinia viruses at 48 hpi and concentrated the TCEs, and then treated HT-29 and COLO 205 cells with the collected supernatants, demonstrating a bystander effect on our J69 reporter line. An increase in td-Tomato fluorescence intensity was observed in conditions treated with filtered supernatants from VV-αCEA:hCD3 (relevant αCEA TCE) but not other virus controls (**Supplementary Figure 8B**). These results indicate that it advantageous to produce TCEs from a large DNA virus, which can be filtered out to study TCE alone effects. In the murine co-culture model using splenocytes, MC38_CEA_ cells showed a greater decrease in cell viability only when infected with VV-αCEA:mCD3 (relevant αCEA TCE) and in the presence of splenocytes (**Figure 2G**). Importantly, there was no effect with VV-CTRL and VV-αCEA:hCD3 (αCEA CTRL TCE), regardless of the absence or presence of murine effector cells. Co-cultures of MC38_WT_ (which lack CEA expression) and murine effector cells showed no difference in tumour cell viability between viruses (**Supplementary Figure 8C**). These data confirm the specificity of our TCEs which target either human or murine T cells.

To further demonstrate the benefit of BEVirs in a 3D model that is more representative of a tumour, we generated spheroids from HT-29 cells stably expressing Nanoluciferase (NLuc) intracellularly. This model provides a simple approach to measure spheroid viability through the quantification of NLuc activity in supernatant, as Nluc is only released upon cancer cell lysis and the luminescent signal can be quantified as a surrogate measure for cancer cell death. Briefly, we grew the spheroids in a methylcellulose matrix for 2 d before infecting with viruses. At 48 hpi, we added PBMCs (E:T = 10:1) or no PBMCs (E:T = 0:1) as a control, as indicated. At 96 hpi, we observed EGFP transgene expression (**Figure 2H**) and quantified the NLuc in the media from each well (**Figure 2I**). There was a significant increase in luminescence for VV-αCEA TCE with PBMCs added, compared to other virus controls and no PBMC conditions (**Figure 2I**). These results agree with our previous findings that VV-αCEA TCE promotes more cancer cell death in the presence of PBMCs in 2D co-cultures (**Figure 2F**). However, our NLuc assay eliminates the possibly of PBMCs acting as a potential confounder in a cell metabolism assay. Interestingly, we could also visualize more rapid breakdown of the spheroid with the virus producing the TCE compared to virus controls, but not with the TCE alone (**Supplementary Figure 8D**). These data suggest that the localized production of TCE from within the infected spheroid is superior to TCE alone delivery.

### Antitumour efficacy of VV encoding TCE in a human xenograft model

**Figure 3:**
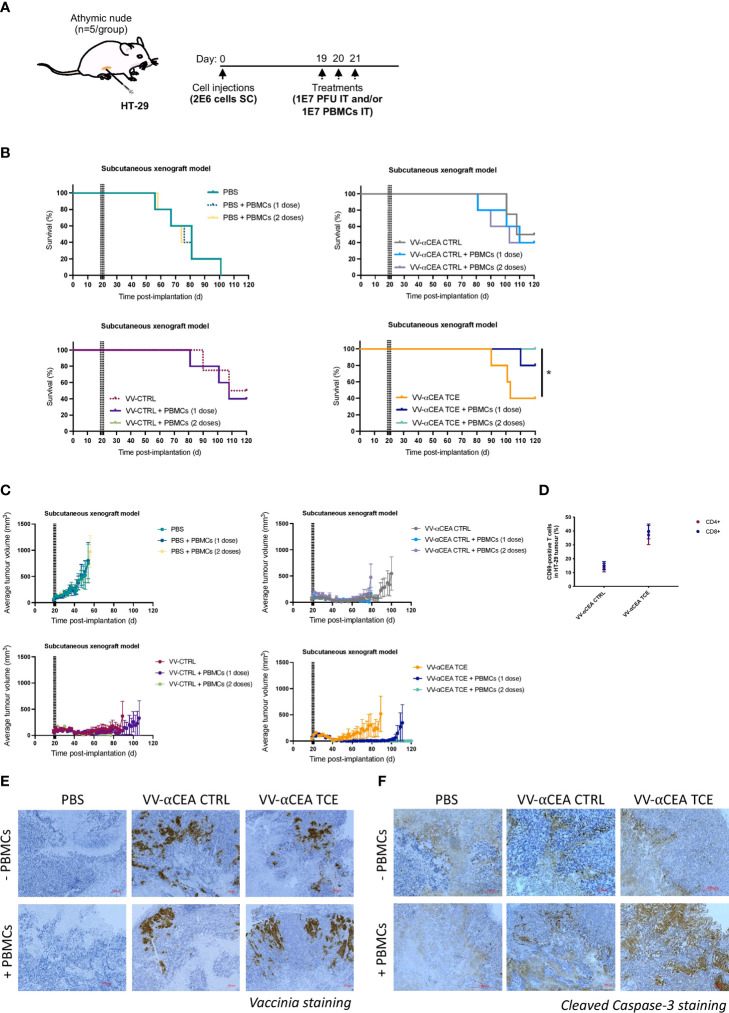
Antitumour efficacy of VV encoding TCE in a human xenograft model. **(A)** Athymic nude mice bearing subcutaneous HT-29 tumours were injected intratumourally with 3 doses of viruses at 1E7 pfu on days 19, 20 and 21. Approximately 5 h after virus injections, mice were also co-injected intratumourally with freshly isolated PBMCs at 1E7 per condition, or PBS control, on day 19 only, or co-injected intratumourally with freshly isolated PBMCs at 1E7 per condition, or PBS, on both days 19 and 21. **(B)** Antitumour efficacies of VV-CTRL, VV-αCEA TCE (αCEA:hCD3), and VV-αCEA CTRL (αCEA:mCD3) viruses were evaluated in combination with one or two doses of PBMCs. Treatment with viruses alone showed significantly prolonged survival (40-50% cures), compared to PBS treatment and regardless of PBMC presence. Highest survival rates were observed in mice treated with VV-αCEA TCE and PBMCs, with 100% survival of mice treated with 3 doses of VV-αCEA TCE and two doses of PBMCs. **(C)** Mice bearing HT-29 tumours co-injected with VV-αCEA TCE and two doses of PBMCs had the most significant decrease in average tumour volumes. **(D)** T cells isolated from HT-29 tumours 24 h after the last dose of injection with VV-αCEA TCE and PBMCs showed an increase in expression of the T cell activation marker CD69, while injection of VV-αCEA CTRL had little effect relative to PBS treatment. **(E)** Vaccinia virus was detected in HT-29 tumours 24 hpi by immunohistochemistry (IHC) in conditions treated with VV-αCEA TCE (αCEA:hCD3) or VV-αCEA CTRL (αCEA:mCD3), but not PBS control. Scale bar = 200 µm. **(F)** Active caspase-3 was predominantly detected in HT-29 tumours 24 hpi by IHC in conditions co-treated with VV-αCEA TCE (αCEA:hCD3) and PBMCs, compared to other virus and no PMBC conditions. Scale bar = 200 µm.

Antitumour efficacies of VV-CTRL, VV-αCEA:hCD3 (relevant αCEA TCE), and VV-αCEA:mCD3 (αCEA CTRL) viruses were evaluated using an *in vivo* human HT-29 xenograft model and intratumoural adoptive transfer of PBMCs. Athymic nude mice bearing subcutaneous HT-29 tumours in each mouse was injected intratumourally with 3 doses of viruses at 1E7 pfu on days 19, 20 and 21 (**Figure 3A**). As these mice lack T cells, we must co-inject PBMCs intratumourally to assess TCE efficacy *in vivo*, as intravenous delivery of PBMCs may lead to graft vs host disease. Thus, approximately 5 h after virus injection, mice were also co-injected intratumourally with freshly isolated PBMCs at 1E7 per condition, or PBS control, on day 19 only, or co-injected intratumourally with freshly isolated PBMCs at 1E7 per condition, or PBS, on both days 19 and 21 (**Figure 3A**). No significant differences were observed in weights of mice after injection with the different viruses (**Supplementary Figure 9A**), and we did not detect any pox lesions on the tails and limbs of these immunocompromised mice compared to mice injected with VV wildtype (**Supplementary Figure 9B**), suggesting off-target toxicity was minimal. Treatment with viruses alone showed significantly prolonged survival (40-50% cures), compared to PBS treatment and regardless of presence or absence of PBMCs (**Figure 3B**). Mice treated with VV-αCEA TCE and PBMCs showed 100% survival when treated with 3 doses of VV-αCEA TCE and two doses of PBMCs (**Figure 3B**). Consistent with these results, we showed that mice bearing HT-29 tumours co-injected with VV-αCEA TCE and PBMCs had a significant decrease in average tumour volumes (**Figure 3C**) and individual tumour volumes (**Supplementary Figure 9C**).

T cells isolated from HT-29 tumours 24 h after the last dose of injection with VV-αCEA TCE and PBMCs showed an increase in expression of the T cell activation marker CD69, while injection of VV-αCEA CTRL had little effect relative to PBS treatment (**Figure 3D**). Immunohistochemistry (IHC) was performed on tumours collected 24 h after virus and PBMCs co-injections, and staining for vaccinia confirmed virus replication within the tumour (**Figure 3E**). As TCEs force the interaction between cancer cells and T cells, leading to release of granzyme B and perforin to activate caspases (**Supplementary Figure 9D**), we also stained for cleaved Caspase-3. We detected an increase in active Caspase-3 in conditions co-treated with VV-αCEA TCE and PBMCs (**Figure 3F**), suggesting that the tumour cells were undergoing apoptosis. One limitation of this model includes the temporary presence of the injected T cells in the tumour, and this highlights the importance of immunocompetent mice to study the effects of TCEs in the TME. Importantly, this *in vivo* study allowed us to demonstrate therapeutic efficacy of vaccinia virus encoding a TCE that targets human CD3 (VV-αCEA:hCD3), which would be necessary for clinical studies in cancer patients.

### Antitumour efficacy of VV encoding TCE in immunocompetent mouse models

**Figure 4:**
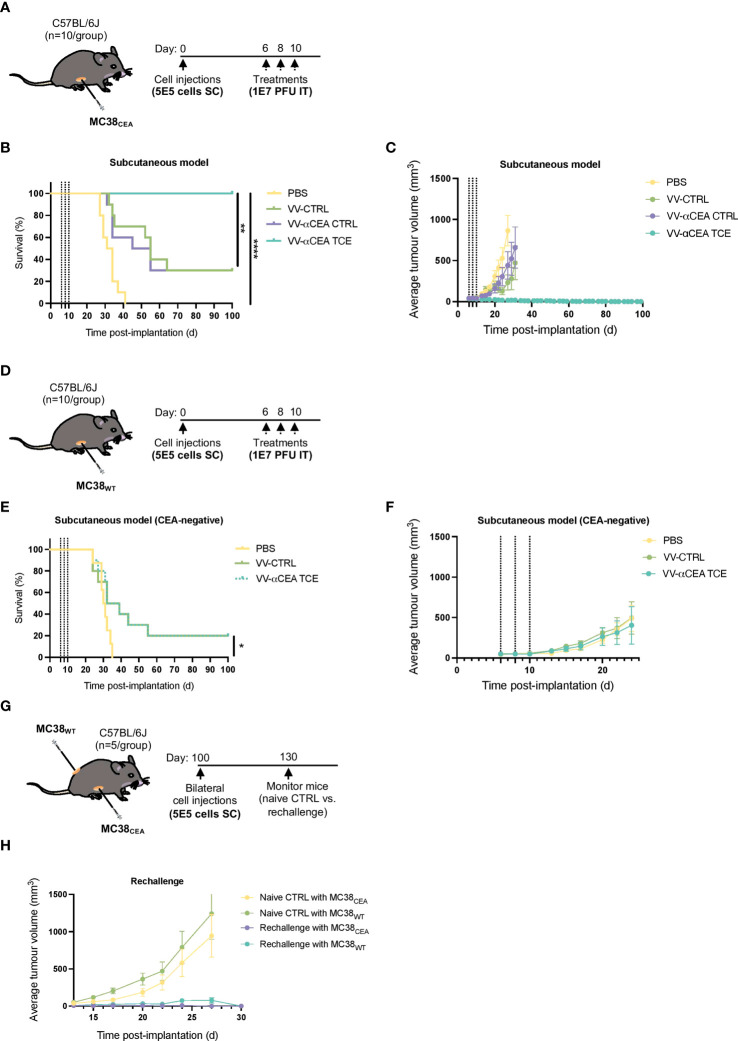
Antitumour efficacy of VV encoding TCE in immunocompetent mouse models leads to cures and immunologic memory. **(A)** C57BL/6J mice bearing subcutaneous MC38_CEA_ tumours were injected intratumourally with 3 doses of viruses at 1E7 pfu, or PBS as control, at days 6, 8 and 10. **(B)** Antitumour efficacies of VV-CTRL, VV-αCEA TCE (CEA:mCD3), and VV-αCEA CTRL (αCEA:hCD3) viruses were assessed in immunocompetent mice. Mice bearing MC38_CEA_ tumours treated with viruses showed significantly prolonged survival compared to PBS controls. Of note, mice treated with VV-αCEA TCE were completely cleared of tumours and 100% cured. **(C)** Mice bearing MC38_CEA_ tumours treated with VV-αCEA TCE (CEA:mCD3) had the most significant decrease in average tumour volumes. **(D)** C57BL/6J mice bearing subcutaneous MC38_WT_ tumours were injected intratumourally with 3 doses of viruses at 1E7 pfu, or PBS as control, at days 6, 8 and 10. **(E)** Antitumour efficacies of VV-CTRL, VV-αCEA TCE (CEA:mCD3), and VV-αCEA CTRL (αCEA:hCD3) viruses were assessed in immunocompetent mice. Mice bearing MC38_WT_ tumours treated with virus showed improved survival, but no difference between VV-CTRL, VV-αCEA TCE (CEA:mCD3) groups. **(F)** Mice bearing MC38_WT_ tumours treated with VV-αCEA TCE (CEA:mCD3) showed no difference in average tumour volumes between VV-CTRL, VV-αCEA TCE (CEA:mCD3) groups. **(G)** Mice cured of MC38_CEA_ after treatment with VV-αCEA TCE were re-challenged with subcutaneous MC38_CEA_ and MC38_WT_ bilateral tumours (5E5 cells) injected at day 100. **(H)** All rechallenged mice rejected MC38_CEA_ and MC38_WT_ tumour engraftment compared to naïve mice controls.

We next investigated the antitumour efficacies of VV-CTRL, VV-αCEA:mCD3 (relevant αCEA TCE), and VV-αCEA:hCD3 (αCEA CTRL) viruses using an *in vivo* immunocompetent C57BL/6J mouse model. Mice bearing subcutaneous MC38_CEA_ or MC38_WT_ tumours were injected intratumourally with 3 doses of viruses at 1E7 pfu, or PBS as control, at days 6, 8 and 10 (**Figures 4A, D**). Mice bearing MC38_CEA_ tumours treated with viruses showed significantly prolonged survival (**Figure 4B**) and decreased tumour volumes (**Figure 4C**; **Supplementary Figure 10A**), compared to PBS controls, with mice treated with VV-αCEA TCE being completely cleared of tumours (**Figure 4B**). No significant differences were observed in weights of mice after injection with the different viruses (**Supplementary Figure 10B**), indicating toxicity was minimal. Mice bearing MC38_WT_ tumours showed improved survival (**Figure 4E**) and delayed tumour growth (**Figure 4F**, **Supplementary Figure 10C**) when treated with virus, compared to PBS, and as predicted, there was no difference in tumour growth or survival between any of the viruses (**Figure 4E**). These findings indicate that the human CEA antigen is required for the TCE-mediated therapeutic effect in these mice. To confirm the curative potential of VV-αCEA TCE, tumour-free mice were re-challenged at day 100 with bilateral subcutaneous MC38_CEA_ and MC38_WT_ tumours (**Figure 4G**). All VV-αCEA TCE treated mice rejected the bilateral tumour engraftment compared to naïve mice controls (**Figure 4H**; **Supplementary Figure 10D**), demonstrating long-lasting immunologic memory against colorectal tumours independent of CEA antigen expression.

**Figure 5:**
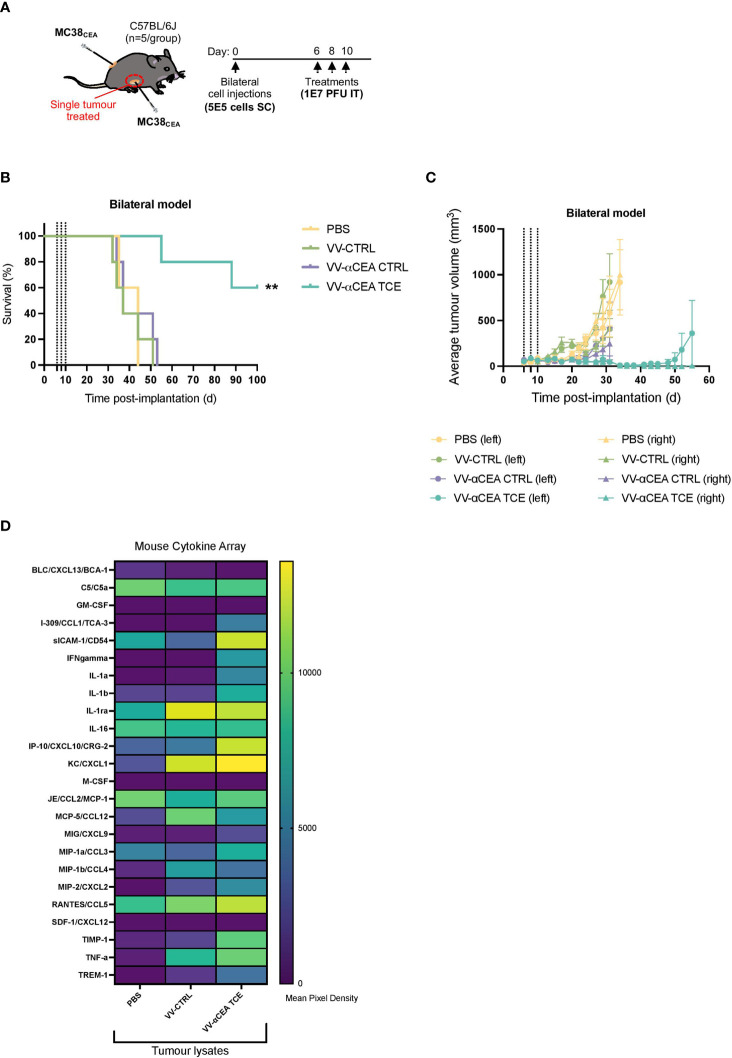
VV encoding TCE leads to abscopal effects and different cytokine profiles in tumours. **(A)** C57BL/6J mice bearing subcutaneous MC38_CEA_ bilateral tumours were injected unilaterally and intratumourally with 3 doses of viruses at 1E7 pfu, or PBS as control, at days 6, 8 and 10. **(B)** Mice treated with VV-αCEA TCE (CEA:mCD3) showed increased survival, and 60% of these mice were cured after unilateral treatment, compared to groups treated with PBS, VV-CTRL or VV-αCEA CTRL (CEA:hCD3). **(C)** Mice treated with VV-αCEA TCE (CEA:mCD3) showed regression of both simultaneously engrafted tumours, compared to groups treated with PBS, VV-CTRL or VV-αCEA CTRL (CEA:hCD3). **(D)** We performed a murine cytokine assay using tumour lysates from subcutaneous MC38_CEA_ tumours in mice treated with VV-αCEA TCE, VV-CTRL or PBS. Of note, differential expression of cytokines was most evident in tumour samples. We detected more abundant levels of sICAM-1, CXCL10, CXCL1, CCL5, TIMP-1 and TNFα in tumours treated with VV-αCEA TCE, compared to tumours treated with VV-CTRL or PBS. We also identified unique signatures after VV-αCEA TCE treatment, including increased levels of CCL1, IFNγ, IL-1a, IL-1b, TIMP-1, and TREM-1.

To determine whether the antitumour immunity was systemic, one of two bilateral MC38_CEA_ tumours in each mouse was intratumourally with 3 doses of viruses at 1E7 pfu, or PBS as control, at days 6, 8 and 10 (**Figure 5A**). Of note, 60% of mice survived after unilateral intratumoural treatment (**Figure 5B**), regression of both simultaneously engrafted tumours was observed (**Figure 5C**; **Supplementary Figure 11A**). These data suggest that a unique profile of chemokines and cytokines may mediate this abscopal effect (**Figure 5A**), as well as the immunologic memory in our *in vivo* rechallenge experiments (**Figure 4H**). To elucidate the *in vivo* production of cytokines upon VV infection, we performed a murine cytokine assay using MC38_CEA_ tumour lysates (**Figure 5D**) and serum (**Supplementary Figure 11B**) from mice treated intratumourally with VV-αCEA TCE, VV-CTRL or PBS (**Figure 5D**). Differential cytokine expression was most pronounced in tumour samples (**Figure 5D**). We detected more abundant levels of sICAM-1, CXCL10, CXCL1, CCL5, TIMP-1 and TNFα in tumours treated with VV-αCEA TCE, compared to tumours treated with VV-CTRL or PBS (**Figure 5D**; **Supplementary Figures 11B–H**). We also identified unique signatures in tumour samples after VV-αCEA TCE treatment, including increased levels of CCL1, IFNγ, IL-1a, IL-1b, TIMP-1, and TREM-1 (**Figure 5D**, **Supplementary Figures 11B–H**). Consistent with other studies (52), we showed that IFNγ can upregulate CEA levels on colorectal cancer cell lines (**Supplementary Figures 11I**).

### Combination viro-immunotherapy for peritoneal carcinomatosis and lung metastases

**Figure 6:**
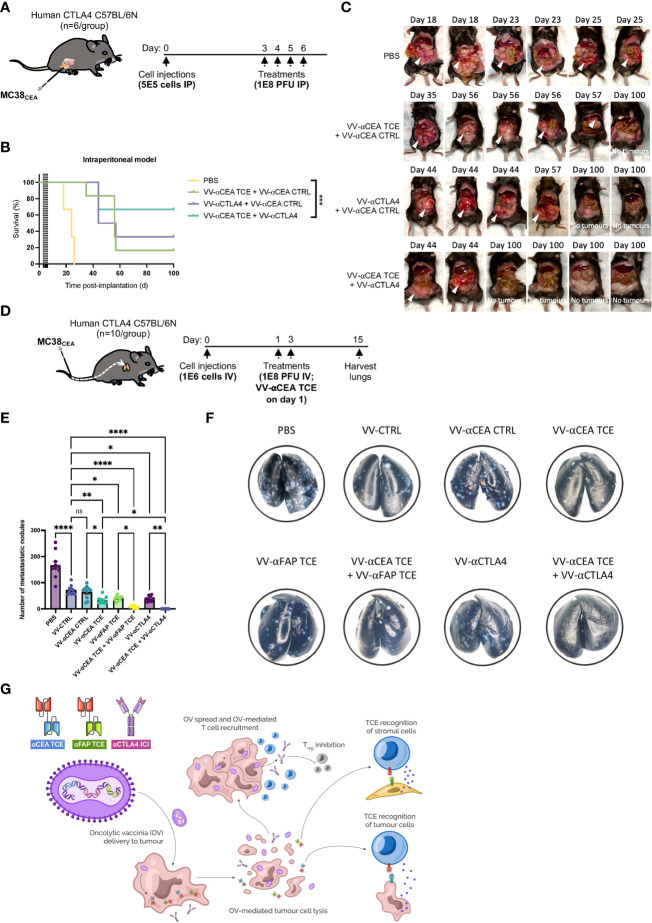
Combination viro-immunotherapy for aggressive CRC peritoneal carcinomatosis and lung metastasis models. **(A)** C57BL/6N mice that express human CTLA4 instead of murine CTLA4 on T cells and bear intraperitoneal MC38_CEA_ tumours, were injected intraperitoneally with 4 doses of viruses at 1E8 pfu, or PBS as control, at days 3, 4, 5 and 6. **(B)** Antitumour efficacies of the following combinations were assessed compared to PBS alone: VV-αCEA TCE (αCEA:mCD3) on days 3 and 5, in combination with VV-αCEA CTRL (αCEA:hCD3) on days 4 and 6; VV encoding αCTLA4 (which recognizes human CTLA4; VV-αCTLA4) on days 3 and 5, in combination with VV-αCEA CTRL (αCEA:hCD3) on days 4 and 6; VV-αCEA TCE (αCEA:mCD3) on days 3 and 5, in combination with VV-αCTLA4 on days 4 and 6. Mice bearing MC38_CEA_ tumours treated with viruses showed significantly prolonged survival in this aggressive peritoneal carcinomatosis model, and 66% of mice were cured after the combination treatment of VV-αCEA TCE and VV-αCTLA4. **(C)** Treated mice were dissected upon endpoint or at day 100 for cured mice to confirm no tumours. **(D)** Following intravenous injection of MC38_CEA_ cells (1E6) in human CTLA4 C57BL/6N mice, we administered 2 doses of viruses at 1E8 pfu, or PBS as control, and harvested the lungs at day 15 for staining. **(E)** In the CRC metastasis model, VV-αCEA TCE, VV-αFAP TCE, and VV-αCTLA4 monotherapies significantly reduced the number of metastatic nodules in the lungs compared to VV-CTRL or VV-αCEA CTRL, and especially PBS control. Combinations of VV-αCEA TCE with either VV-αFAP TCE or VV-αCTLA4 demonstrated synergy in further decreasing the number of lung metastases. Some of the lungs of the mice treated with VV-αCEA TCE and VV-αCTLA4 were completely cleared of metastases. Results show number of metastatic nodules ± SEM; One-way ANOVA. **(F)** Images of metastases (white spots) in lungs harvested from mice at day15, after being inflated with india ink and fixed. **(G)** Schematic illustrating the combination of viro-immunotherapies targeting CEA on cancer cells and FAP on stromal cell populations (CAFs) by TCE, and T_regs_ through immune checkpoint inhibitor (ICI) αCTLA4 to boost T cell responses for the treatment of CRC.

Depletion of T_reg_ cells or elimination of stromal cells to potentiate endogenous antitumour immune responses is an appealing strategy in the context of colorectal cancers (15, 16). Combinations of TCEs targeting CEA and/or FAP on malignant CRC tumours with immune checkpoint inhibitors, such as αCTLA4 (48, 49), may be crucial to maintain the acute efficacy of TCEs in aggressive models of peritoneal carcinomatosis and CRC metastases to lungs. Using immunocompetent C57BL/6N mice that express human CTLA4 on T cell populations, we evaluated the antitumour efficacies of VV-αCEA:mCD3 (relevant αCEA TCE), in combination with VV-αCEA:hCD3 (αCEA CTRL) or VV encoding αCTLA4 (which recognizes human CTLA4; VV-αCTLA4), compared to VV-αCEA:hCD3 (αCEA TCE) with αCTLA4, or PBS control. Mice bearing intraperitoneal MC38_CEA_ tumours were injected intraperitoneally with 4 doses of viruses at 1E8 pfu, or PBS as control, at days 3, 4, 5 and 6 (**Figure 6A**). Of note, this is highly aggressive model of peritoneal carcinomatosis, whereby tumours grow upon the gut (**Supplementary Figure 12A**) and mice are generally euthanized before day 25 (**Supplementary Figures 12B–D**), due to abdominal distension and respiratory distress. We first assessed the combination of αCTLA4 with VV-αCEA TCE which increased survival of mice (**Supplementary Figures 12B–D**). We then encoded αCTLA4 into vaccinia virus to allow for combination viro-immunotherapy. Mice bearing MC38_CEA_ tumours treated with viruses showed significantly prolonged survival (**Figure 6B**) and 66% of mice were cured after the combination treatment of VV-αCEA TCE and VV-αCTLA4 (**Figures 4B, C**; **Supplementary Figure 12A**).

As previously mentioned, immunosuppressive stromal cells can act as a barrier to immunotherapies. Populations of FAP-positive CAFs have been successfully targeted by TCEs encoded in VV or AdV (26, 53). Using our established pipeline (**Supplementary Figure 1B**), we designed and characterized a TCE that recognizes murine FAP that is not expressed by MC38 cells *in vitro*, but is abundantly expressed by the stromal cells in the TME of MC38 tumours *in vivo* (54). We encoded the αFAP TCE into vaccinia virus (VV-αFAP TCE) as previously described (**Supplementary Figure 5C**) and demonstrated *in vivo* efficacy and modest prolonged survival in a subcutaneous MC38_WT_ tumour model in C57BL/6J mice (**Supplementary Figure 12E**). We further studied the effect of our combination strategies in an aggressive CRC metastasis model to the lungs. Following intravenous injection of MC38_CEA_ cells in human CTLA4 C57BL/6N mice, we administered 2 doses of viruses at 1E8 pfu, or PBS as control, and harvested the lungs at day 15 for staining (**Figure 6D**). We showed that VV-αCEA TCE, VV-αFAP TCE, and VV-αCTLA4 monotherapies significantly reduced the number of metastatic nodules in the lungs of C57BL/6N mice, compared to VV-CTRL or VV-αCEA CTRL controls, and especially PBS control (**Figures 6E, F**). Single-dose combinations of VV-αCEA TCE with either VV-αFAP TCE or VV-αCTLA4 demonstrated synergy in further decreasing the number of lung metastases (**Figures 6E, F**). In fact, some of the lungs of the mice treated with VV-αCEA TCE and VV-αCTLA4 were completely cleared of metastases, emphasizing the importance of our combination viro-immunotherapies for the treatment of CRC (**Figure 6G**).

## Discussion

Cancer immunotherapy is revolutionizing clinical medicine, with a subset of MSI-H/dMMR CRCs and other tumour types, such as melanoma and non-small cell lung cancer, showing durable clinical responses to immune intervention (3, 4, 10). One of the most successful approaches to treat challenging tumours has been immune checkpoint inhibition (3, 4, 10, 49), often combined with several immunostimulatory strategies to drive additive or synergistic therapeutic effects (40–47). Despite these successes, most CRCs avoid immune recognition through loss or downregulation of HLA, which may decrease recruitment of antitumour T cells in the TME and further contribute to immunosuppression by T_regs_ and stromal cell populations (15, 16). Tumours without functional HLA are consequently not susceptible to T cell vaccines or immune checkpoint inhibition. TCE therapy, in contrast, circumvents the need for antigen presentation by HLA, thereby facilitating the interaction between T cells and tumour cells expressing unique cell-surface proteins on malignant cell types, regardless of the mutations that the tumour carries (7). The orchestration of potent T cell responses against CRCs is necessary for effective antitumour immunity, suggesting that the use of TCEs may be more advantageous than bispecific engagers that interact with NK cells (55) or macrophages (56). As such, we have designed TCEs to target CEA (**Figures 1A, B**; **Supplementary Figure 1A**), a cell surface protein abundantly expressed on colorectal tumour cells, especially after treatment with standard of care 5-FU chemotherapy (**Supplementary Figure 2E**), or the FAP antigen, expressed on CAF populations, as a two-pronged immunostimulatory attack on CRCs to overcome tumour heterogeneity and stromal barriers in the TME of CRC, respectively. We have shown that TCEs robustly activate CD4 and CD8-positive T cells in co-culture models with CRC cell lines and patient tumour-derived cell lines that express CEA (**Figures 1C–F**), but *in vivo* efficacy is hindered by lack of TCE retention in the tumour and short TCE half-life (**Supplementary Figure 3D**), as demonstrated by HiBiT bioluminescent TCE-tagging technology (**Supplementary Figures 3A–C**). These findings are consistent with previous studies that have identified delivery of TCEs to solid tumours as a significant obstacle in preclinical models and cancer patients (7).

To overcome TCE limitations and challenges, we have engineered BEVirs using a novel tumour selective VV Copenhagen platform (ClinicalTrials.gov: NCT04301011) for *in situ* tumour delivery of these immunomodulatory molecules (**Figure 2E**; **Supplementary Figure 5C**). VVs are more than cancer-lysing and immune-activating biotherapeutics, as they are also self-limiting gene delivery systems that can specifically express anticancer therapeutics in the TME (5). Although previous clinical trials have demonstrated safety for modified VVs in CRC patients (38, 39), other virus strains have been overly attenuated and/or lack potency as an oncolytic for human tumours. As further rationale to use our VV platform in the context of CRC, we have shown that our modified VV can outperform other oncolytic viruses by infecting both human and murine cancer spheroid models (**Figures 1G, H**). We have also shown that TCE combination with VV provides better efficacy than TCEs alone (**Figures 2C, D**), likely due to VV creating an inflamed tumour and recruiting T cells (**Figures 2A, B**).

We characterized virus-mediated TCE-secretion (**Supplementary Figures 5D, E**, **6A–D**), TCE specificity and functionality from infected colorectal cancer cells (**Supplementary Figure 8B**), as well as TCE cytotoxicity in co-cultures with or without T cells (**Figures 2F, G**). VVs engineered to express TCEs under a late promotor did not show a decrease in replication or oncolytic capabilities in murine and human cell lines and infected cells showed secretion of TCEs (**Supplementary Figures 7A–J**). Furthermore, TCE-encoding VVs reduced cell viability in murine and human spheroid CRC models (**Figures 2H, I**), but only in the presence of the target antigen and when co-cultured with the appropriate effector cells.

We have designed TCEs targeting either human or murine CD3 (**Figure 1A**; **Supplementary Figure 1A**) to evaluate the therapeutic efficacy of VVs encoding TCEs in both a human xenograft model bearing HT-29 tumours with transfer of human PBMCs (**Figures 3A–C**) and an immunocompetent mouse model bearing MC38_CEA_ tumours (**Figures 4A–C**), respectively. Importantly, we have demonstrated that therapeutic efficacy of VV in solid tumour models can be enhanced by encoding TCEs into the virus, consistent with other oncolytic virus-TCE studies (24–33). As predicted, there were no significant differences in therapeutic effects between viruses in the MC38_WT_ CEA-negative model (**Figures 4D–F**), confirming that expression of the target antigen is needed for TCEs to be effective. Of note, we have demonstrated BEVir safety in both xenograft and syngeneic mouse models (**Supplementary Figures 9A, B**, **10B**). In the immunocompetent mice bearing MC38_CEA_ tumours, injection of VV-αCEA TCE resulted in full clearance of the subcutaneous tumours (**Figures 4A–C**) and provided mice with protective antitumour immunity upon re-challenge (**Figures 4G, H**). In our xenograft model, HT-29 tumours were also cured in the presence of VV-αCEA TCE and co-injection with PBMCs (**Figures 3A–C**). T cells within the tumour were activated in the tumours in the presence of VV-αCEA TCE, as shown by flow cytometry and IHC (**Figures 3D–F**). Our data suggest that potential CEA shedding did not impact therapeutic efficacy in our models, consistent with other TCE studies (18, 19). Since oncolytic viruses expressing transgenes have been approved for cancer patients and new platforms are moving into clinical trials (5), TCEs provide a promising new approach for combination with oncolytic viruses in the clinical setting. Viral production of TCEs in the tumour may overcome the short half-life and off-target toxicity observed with systemic IV administration of TCEs (18, 19).

A plethora of immunostimulants, including damage-associated molecular patterns, pathogen-associated molecular patterns, and cytokines, are released upon virus-mediated cell lysis and contribute antitumour responses (5, 24). Combining oncolytic viruses with TCEs provides an additional mechanism of cancer cell death, mediated by perforin and granzyme B release from T cells (**Figure 9D**), and creates a bystander killing effect in the TME. Our data suggest that a different profile of cytokines upon VV-αCEA TCE infection may contribute to the pro-inflammatory and immune effects driven by T cells in the tumour microenvironment to provide long-lasting immunity (**Figures 4G, H**) and abscopal effects (**Figures 5A–C**). The unique cytokines identified after VV-αCEA TCE treatment *in vivo* (**Figure 5D**) may facilitate overcoming immune suppression in the TME, recruit additional T cells into the tumour or further boost T cell activation. More specifically, we have detected increased levels of sICAM-1, CXCL10, CXCL1, CCL5, TIMP-1 and TNFα in tumours treated with VV-αCEA TCE, compared to tumours treated with VV-CTRL or PBS (**Figure 5D**, **Supplementary Figures 11B–H**). Interestingly, we also identified unique signatures after VV-αCEA TCE treatment, including increased levels of CCL1, IFNγ, IL-1a, IL-1b, TIMP-1, and TREM-1 (**Figure 5D**; **Supplementary Figures 11B–H**). These results are consistent with previously studies (31, 33) that have characterized the role of IFNγ and TNFα in mediating TCE effects in the TME. In addition, we and others (52) have shown that IFNγ can upregulate CEA levels on colorectal cancer cell lines (**Supplementary Figure 11I**), similarly to the effect of 5-FU boosting CEA and TCE binding (**Supplementary Figures 2E, F**). These findings suggest that a feedforward cycle may exist in the TME, whereby VV-αCEA TCE treatment induces IFNγ production, elevating CEA levels and sensitizing CEA-positive tumour cells to TCE therapy. Interestingly, IL-1a and IL-1b can be secreted by macrophages and interact with receptors on FAP+ CAFs, promoting the expression of fibroblast ECM-related genes (15). These results may suggest that combination of BEVirs targeting both CEA and FAP may act synergistically by inhibiting these immunosuppressive interactions in the TME. We have therefore assessed the efficacy of VV-αFAP TCE in MC38_WT_ tumours, which produce a FAP-positive stroma *in vivo (* 54), with the intention of combining the treatment with VV-αCEA TCE. We have shown that VV-αFAP TCE only modestly increases survival of mice bearing tumours (**Supplementary Figure 12E**), compared to VV-αCEA TCE treatment, suggesting that VV may replicate and kill CAF populations in the TME effectively (35), even in the absence of TCE.

Combining oncolytic viruses with immune checkpoint inhibition shows promising results in clinical trials (5), and studies have shown that VV infection may increase expression of exhaustion markers. As we did not detect increases in PD-1 levels on T cells upon infection of tumours (**Figure 2B**), we instead focused on elucidating the potential immunosuppressive role of CTLA4 in the TME due to its relevance in CRC. Interestingly, elevated circulating Treg frequency correlated with reduced activity of TCE therapy in cancer patients (57). Others have shown that depletion of all CD4+ antitumour T cells and T_regs_ significantly increased TCE efficacy and hypothesized that the depletion impaired the ability of T_regs_ to inhibit CD8+ T cell cytolytic activity (42). Thus, we established combination regimens of VV-αCEA TCE and VV-αCTLA4 for the treatment of aggressive colorectal peritoneal carcinomatosis (**Supplementary Figure 12A**). After assessing the combination of αCTLA4 with VV-αCEA TCE (**Supplementary Figures 12B–D**), we encoded αCTLA4 into vaccinia virus to allow for combination viro-immunotherapy and localized delivery of αCTLA4 to reduce potential systemic toxicities. Mice bearing MC38_CEA_ intraperitoneal tumours treated with viruses showed significantly prolonged survival (**Figure 6B**) and 66% of mice were cured after the combination treatment of VV-αCEA TCE and VV-αCTLA4 (**Figures 4B, C**; **Supplementary Figure 10A**). We also observed a significant reduction in lung metastases of colorectal tumours through intravenous delivery of our oncolytic virus driven T-cell based combination immunotherapy to target CEA-positive colorectal tumours, FAP-positive stromal cells and/or CTLA4-positive T_reg_ cells in the tumour microenvironment. Combinations of VV-αCEA TCE with either VV-αFAP TCE or VV-αCTLA4 demonstrated synergy in further decreasing the number of lung metastases (**Figures 6E, F**). In fact, some of the lungs of the mice treated with VV-αCEA TCE and VV-αCTLA4 were completely cleared of metastases, emphasizing the importance of enhancing immune-payload delivery *via* oncolytic virus to boost T cell responses in tumours and combining viro-immunotherapies for the treatment of CRC (**Figure 6G**).

## Materials and methods

### Cell culture

HT-29, SW620, COLO 205, HCT 116, HCT15, BxPC-3, MIA PaCa-2, HEK293T, U2OS, MCF7, A549, Vero, and U87MG cells (ATCC; VA, USA) were cultured in Dulbecco’s Modified Eagle Medium/DMEM (GE Healthcare Life Sciences; ON, CAN) or Roswell Park Memorial Institute/RPMI 1640 Medium (Gibco; MA, USA), supplemented with 10% foetal bovine serum/FBS (Gibco). Adeno-X 293 cells were gifted by Dr. Nikolas Martin (Ottawa Hospital Research Institute/OHRI, Canada). MC38_WT_ and MC38 cells expressing human CEA/MC38_CEA_ were gifted from the Dr. Guy Ungerechts (OHRI, Canada). Cells were maintained in a humidified atmosphere at 37°C in 5% CO_2_. For virus inoculation as well as TCE-pcDNA, VV-TCE plasmid or TCE detection in supernatant, cells were maintained in DMEM without FBS. Cell lines derived from patient tumours and cells co-cultured with effector cells were grown in RPMI media supplemented with 10% FBS and 1% (volume/volume) penicillin/streptomycin (Gibco). Co-cultures were maintained in a humidified atmosphere at 37°C in 5% CO_2_. Cells were routinely tested for potential mycoplasma contamination by PCR (e-Myo VALiD Detection Kit, 25239, LiliF Diagnostics; South Korea) and remained mycoplasma-free.

### Isolation of peripheral blood mononuclear cells and murine splenocytes

Human peripheral blood mononuclear cells (PBMCs) were provided by the Ottawa Hospital Research Institute (OHRI) Cancer Center Blood Clinic and isolated using density gradient centrifugation. Blood was diluted one in two with PBS and overlaid on Ficoll (1,079 g/ml, Ficoll-Paque Plus; GE Healthcare Life Sciences). Overlay was centrifugated at 400 g for 30 min at 20°C with low acceleration and deceleration. PBMCs were collected and washed twice with PBS (centrifugated at 500 g for 5 min), counted by ViCell and resuspended in RPMI supplemented with 10% FBS and 1% penicillin/streptomycin. Splenocytes were isolated from mouse spleens using 40 µM Falcon cell strainers (VWR; ON, CAN). Cell strainers were washed with RPMI before centrifugation at 500 g for 5 min. Cells were resuspended in Ammonium-Chloride-Potassium lysis buffer (ACK, A1049201; ThermoFisher Scientific, MA, USA) and incubated at room temperature for 5 min. Cells were washed twice with PBS, counted by ViCell and resuspended in RPMI supplemented with 10% FBS and 1% penicillin/streptomycin.

### Design of TCE vectors

αCEA TCE constructs were derived from anti-human CD3 (OKT3) or anti-murine CD3 (145-2C11) scFv sequences (EMBL-EBI database and IMGT database, respectively). Anti-CD3 sequences were optimized for codon usage by *Mus musculus*. A flexible glycine-serine (GS) linker was used to connect two scFv fragments. TCE sequences contain a Kozak and Igκ leader sequence for secretion. For detection of TCEs a N-terminal Flag-tag and a C-terminal His-tag were included. Alternatively, some TCEs had a N-terminal HiBiT-tag and a C-terminal His-tag, or a N-terminal His-tag and a C-terminal HiBiT-tag. αFAP TCE constructs were derived from anti-murine CD3 (145-2C11) and anti-FAP scFv sequences, and αCTLA4 was designed based on ipilimumab sequences. Plasmids were synthesized (GenScript, NJ, USA).

### TCE expression

TCE-pcDNA was transfected into HEK293T cells cultured in a 15cm² plate using 20 µg DNA and 60 µl lipofectamine 2000 (DNA:lipofectamine 2000, ratio 1:3), according to the manufacturer’s instructions (Life Technologies, ON, CAN), in OptiMEM (ThermoFisher Scientificfor 6 h at 37°C in 5% CO_2_. After incubation the transfection mix was replaced with serum free DMEM and cells incubated for 48 h at 37°C in 5% CO2. For concentration of TCEs, supernatant from transfected cells was collected and centrifugated at 400 g for 5 min at room temperature to clear cell debris. Supernatants were transferred to centrifugal concentrators (Amicon Ultra-15 Centrifugal Filter Units 10 kDa, UFC901024; EMD Millipore, MA, USA) with a 10 kDa molecular weight cutoff and centrifugated at 4500 g for 30 min at 4°C. Concentrates were aliquoted and stored before use at -80°C. TCE quantification performed using a pre coated anti-His ELISA (His-Tag Detection ELISA kit 96 strip wells, 10012445; Cayman Chemical, MI, USA) according to manufacturer’s protocol. For TCE detection upon viral infection, cells were inoculated with virus in serum free DMEM for 2 h at 37°C in 5% CO_2_. Inoculation media was replaced with serum free DMEM and supernatants collected at indicated time points by centrifugation at 400 g for 5 min at room temperature. Supernatants were stored at -20°C before using. For concentrating TCEs from virally infected cells supernatants were first filtered twice using 0.22 µM filters before loading into the centrifugal concentrators with 10 kDa cutoff (EMD Millipore).

### Generation of TCE expressing vaccinia virus

αCEA and αFAP TCE transgenes (from TCEpcDNA plasmids) were inserted into a VV expression backbone (VV-TCE plasmid) with homology arms for the vaccinia B14R locus/3p large deletion of the virus, using standard DNA cloning techniques (restriction digest with XhoI and NotI NEB enzymes and ligation), and sequence verified. αCTLA4 + eGFP was inserted into the 5p large deletion of the virus under a pEarly promoter. U2OS cells were infected for 2 h with Copenhagen vaccinia virus (VV-CTRL; Cop5p-3p-, patent publication 20220056480 and backbone of VV in ClinicalTrials.gov: NCT04301011) at a MOI of 0.01, after which media was removed and cells were transfected with 1 µg of DNA (DNA:lipofectamine 2000 ratio 1:3). After 2 h, the transfection mix is removed and replaced with DMEM supplemented with 10% FBS. TCE transgenes insert into the virus genome through recombination. Cells were incubated for 48 h at 37°C in 5% CO_2_ and checked for expression of eGFP. Cells positive for eGFP were collected and freeze/thawed 3x and sonicated 2x. Collected virus was serially diluted and used to infect new U2OS cells. After infection U2OS cells were cultured with overlay medium (1:1 of 3% CMC and 2x DMEM + FBS) for 48 h at 37°C in 5% CO_2_. eGFP positive plaques were picked using sterile pipette tips, freeze/thawed 3x, and used to again infect U2OS cells. This process was repeated until pure virus was produced, validated using DNA sequencing.

### Oncolytic virus production and titration

For production of vaccinia virus, HeLa cells in 850 cm² roller bottles were infected with virus at a MOI of 0.03 without removing the inoculation media. Cells were cultured for 72 h at 37°C in 5% CO_2_ or until sufficient cytopathic effect was observed and then pelleted and resuspended in 1 mM Tris with a pH of 9.0. Cells were freeze/thawed 3x and centrifugated at 2000 rpm for 10 min at room temperature. Supernatant was collected and overlaid onto 36% sucrose cushions before centrifugation at 11500 rpm for 1 h and 30 min at 4°C. Viral pellets resuspended in 1 ml of 1 mM Tris and stored at -80°C. Viral titers were determined by titration. Virus stocks were serially diluted ten-fold and dilutions used to infect U2OS cells in 12-well plates. After incubation for 2 h at 37°C in 5% CO_2_ media was replaced with overlay medium (1:1 of 3% CMC and 2x DMEM + FBS) and incubated for 48 h at 37°C in 5% CO_2_. After 48 h, plaques were stained with crystal violet and quantified to calculate plaque forming units per ml.

VSVΔ51 encoding eGFP was cultured and titered in Vero cells as previously described (58). HSV encoding eGFP was a gift from Dr. Karen Mossman (McMaster University, Canada) and it was also cultured and titered in Vero cells, using previously described methods (59). MeV encoding eGFP (Schwarz strain) was a gift from Dr. Guy Ungerechts (Ottawa Hospital Research Institute, Canada). AdV (AdRP3089 strain) was gifted by Dr. Robin Parkes (Ottawa Hospital Research Institute, Canada) and has been described previously (60). It is a replication-competent AdV type 5 that contains a monomeric RFP coding sequence with an upstream splice acceptor site replacing the viral early-region 3 (E3) region, which places RFP expression under the control of the viral major late promoter. Thus, AdRP3089 only expresses appreciable levels of RFP late during the virus lifecycle and only if the virus undergoes active replication. AdRP3089 was propagated on Adeno-X 293 cells and purified by cesium chloride buoyant density centrifugation and titered using standard techniques (61).

### siRNA treatment

To assess differences in virus replication in the presence of CEA knockdown or Mock knockdown, siRNA targeting CEA (ON-TARGETplus Human CEACAM5 siRNA, L-004567-01-0005; GE Healthcare Life Sciences) or a non-targeting control/NTC (ON-TARGETplus Non-targeting Pool siRNA, D-001810-10-05; GE Healthcare Life Sciences) was used according to manufacturer instructions, along with buffer (5x siRNA Buffer, B-002000-UB-100; GE Healthcare Life Sciences) and DharmaFECT 1 Transfection reagents (DharmaFECT 1, T-2001-01; GE Healthcare Life Sciences). Briefly, transfections of cells were performed in 12-well plates using 5 µl of 5 µM siRNA/well + 1.6 µl of DharmaFECT/well. Cells were transfected 48 h prior to virus treatment and then collected for viral titration at 48 hpi.

### Immunoblotting

Whole cell lysates were collected for validation of TCE expression by lysing cells on ice after 2x washes with PBS and then resuspending in radioimmunoprecipitation assay buffer (RIPA buffer, 89901; ThermoFisher Scientific) and 1x protease/phosphatase inhibitors (PPI 100x, 5872S; Cell Signaling Technology). Protein concentrations were determined by bicinchoninic acid assay (BCA kit, 23227; ThermoFisher Scientific). Samples were mixed with 1x NuPage LDS sample buffer (LDS 4x, NP0007; ThermoFisher Scientific) and loaded into precast SDS-PAGE gels (NP0322BOX; ThermoFisher Scientific). Immunoblotting was performed with equal amounts of protein for whole cell lysates, supernatant or concentrate samples. Samples were denatured using boiling only, since addition of reducing agents diminished detection of TCEs by immunoblotting. Samples were run with NuPAGE 1x MOPS running buffer (20x MOPS; ThermoFisher Scientific) and transferred onto nitrocellulose membrane, as previously described (46). His-tagged TCEs were detected with mouse anti-His antibody (1:1000, ab18184; Abcam). CEA was detected by mouse anti-human CEACAM5 (1:1000; 2383S, Cell Signaling Technology). Vaccinia virus was detected by a rabbit polyclonal antibody to vaccinia virus (1:1000; LS-C103289, LSBio). For samples with a HiBiT tag, 1x passive lysis buffer (Luciferase Assay System Passive Lysis 5x Buffer, E1941; Promega, WI, USA) was used to harvest whole cell lysates. To detect the HiBiT tag, the nitrocellulose membrane was incubated for 30 min with LgBiT (1 µl of LgBiT/ml of PBS; Promega) and then exposed using nanoglo (5 µL/ml of PBS) substrate (Nano-Glo Luciferase Assay System, N1120; Promega). β-Actin (1:1000; 13E5, Cell Signaling Technology) was used as a loading control. Total protein was assessed by ponceau staining (Ponceau S solution, P7170-1L; Sigma-Aldrich, MO, USA). Human IFNγ 0.1 ng/ml (recombinant IFN-Gamma human, SRP3058; Sigma-Aldrich) and 5-FU (5-Fluorouracil, F6627; Sigma-Aldrich) at indicated concentrations were used to treat cells for 24 h to determine any changes in CEA levels by immunoblotting. Following primary antibody overnight incubations, immunoblots were probed for 1 h with HRP-coupled anti-mouse or anti-rabbit antibodies (1:3000, Cell Signaling Technology) and then imaged on a Bio-Rad ChemiDoc. Densitometry was performed using Fiji/ImageJ software (Freeware; NIH, Bethesda, MD).

### Flow cytometry

Flow cytometry analyses were performed using a BD LSRFortessa flow cytometer (BD Biosciences; CA, USA) and FlowJo v10 software (Treestar Inc., OR, USA) was used for data analysis. For gating, fluorescent-minus-one and compensation controls were prepared (Onecomp eBeads Compensation, 01111142; ThermoFisher Scientific). For *in vivo* experiments, treated MC38_WT_ tumours were dissected, and single cells were obtained using the Tumour Dissociation Kit, mouse (130-096-730, Miltenyi Biotec, Bergisch Gladbach, Germany), according to manufacturer’s protocol. Dead cells were excluded using Fixable Viability Stain 510 (1:1000, 564406; BD Bioscience) and Fc receptors were blocked using CD16/CD32 rat anti-mouse (1:100, Clone: 2.4G2, 553142; BD Bioscience). Cells were stained with CD45 rat anti-mouse (1:1000, Clone: 30 F11, BV786, 564225; BD Bioscience), CD3 hamster anti-mouse (1:300, Clone: 500A2, AF700, 557984; BD Bioscience), CD4 rat anti-mouse (1:1000, Clone: RM4 5, V450, 560468; BD Bioscience), CD8a rat anti-mouse (1:100, Clone: 53-6.7, PE-CF594, 562283; BD Bioscience), CD49b rat anti-mouse (1:100, Clone: DX5, FITC, 553857; BD Bioscience), CD69 hamster anti-mouse (1:100, Clone: H1.2F3, BV605, 563290; BD Bioscience), CD25 rat anti-mouse (1:100, Clone: PC61.5, PE, 12-0251-82; ThermoFisher Scientific), and CD279 hamster anti-mouse (1:100, Clone: J43, APC, 562671; BD Bioscience). All cells were fixed using 1% PFA before analysis. See gating strategy in supplementary data.

CD69 expression was determined for co-cultured PBMCs and TILs. For co-cultured PBMCs, freshly isolated PBMCs were co-cultured with 1000 ng/ml αCEA:hCD3 or αCEA:mCD3 and with or without HT-29 cells. After incubating for 24 h at 37°C in 5% CO_2_ all cells were collected, washed and stained. For TILs, HT-29 tumours were dissected, and single cells were obtained using the Tumour Dissociation Kit, human (130-095-929, Miltenyi Biotec, Bergisch Gladbach, Germany), according to manufacturer’s protocol and stained with antibodies. For both, the following antibodies were used to determine different cell populations: CD3ϵ mouse anti-human (1:100, Clone: UCHT1, PE-Cyanine7, 25-0038-42; ThermoFisher Scientific), CD4 mouse anti-human (1:100, Clone: RPA-T4, PE, 36-102-1464; ThermoFisher Scientific), CD8 mouse anti-human (1:100; Clone: RPA-T8, APC, 555369, BD Bioscience) and CD69 mouse anti-human (1:100, Clone: 5N50, BV421, 562884, BD Bioscience). All cells were fixed using 1% PFA before analysis.

### *In vitro* co-culture cell viability assays

TCE-mediated T cell cytotoxicity induced by TCEs alone or by TCE expressing VV was determined by resazurin assay. The metabolic activity of the cells was assessed using resazurin sodium salt (R12204; ThermoFisher Scientific), according to the manufacturer’s protocol. Treated and/or infected cells were administered 10% (v/v, final) resazurin in each well and incubated for 2–4 h, depending on the cell line, or overnight for spheroids. Fluorescence was measured at 590 nm upon excitation at 530 nm using a BioTek Microplate Reader (BioTek, Winooski, VT, USA). Briefly, freshly isolated effector cells (PBMCs or splenocytes) were co-cultured with target cells (HT-29, COLO205, MC38_WT_ and MC38_CEA_ cells) (Effector : Target cell ratio is 5:1) in a 24-well plate or a flat-bottom 96-well plate with the addition of media alone, TCEs alone or virus. In short, 200,000 (24-well plate) or 15,000 (96-well plate) target cells were seeded 24 h prior. Virus infections were performed at indicated MOIs to cells in serum free media. After 2 h media was removed and replaced with effector cells (E:T = 5:1) in RPMI supplemented with 10% FBS and 1% (v/v) penicillin/streptomycin. For co-cultures with TCEs alone the TCEs were added at indicated concentrations together with effector cells. After incubation at 37°C in 5% CO_2_ for 48 h (or indicated otherwise) cells were verified for EGFP expression using an EVOS fluorescent microscope (ThermoFisher Scientific). To also visualize cells, HT-29 were transduced with lentivirus (62) to express Azurite (pLV-Azurite, Plasmid#36086; Addgene, MA, USA) and PBMCs/splenocytes stained red according to manufacturer instructions (CellTracker Red CMTPX Dye, C34552; ThermoFisher Scientific).

### TCE-cancer cell binding assays

For the TCE binding assay, cells were placed on ice and treated with His-tagged TCEs for 1 h prior to washing away excess unbound TCEs and measuring the absorbance of TCEs bound to the cell surface of live cells after incubating for 30 min using an Alexa-conjugated His antibody (6x-His Tag Monoclonal Antibody Alexa Fluor 647, MA1-21315-A647; ThermoFisher Scientific) to quantify TCE attachment to cell-surface CEA on cancer cells.

### Spheroid infections and co-culture assays

Cell lines were seeded at 1E4 cells per well in 0.12% methyl cellulose (Methyl Cellulose, M7027; Sigma-Aldrich) in media in repellent 96-well plates (Greiner Bio-One 96W Cell-Repellent Plate, PS, Sterile, Round U Bottom, Clear, w/Lid, 32/cs – GBO, 650970; ThermoFisher Scientific). At 48 h, spheroids were infected with indicated viruses at an MOI of 1. For VV-CTRL, VSVΔ51, MeV, HSV and AdV infected cells, GFP or RFP was imaged using a Cellomics ArrayScan platform. Exposure times and intensity thresholds were optimized for each scan to minimize background in uninfected control wells. Cell viability was assessed by resazurin assay at indicated times.

HT-29 cells were transduced with lentivirus (62) to express non-secreted Nanoluciferase (NLuc). The NLuc construct (pcDNA3.1-Nanoluc-ccdB, Plasmid#87070; Addgene) was inserted into a pLenti plasmid (pLenti PGK Blast V5-LUC, Plasmid#19166; Addgene) by standard cloning techniques (restriction digest with SalI and XbaI NEB enzymes and ligation)). For spheroid co-cultures, HT-29 cells that express intracellular NLuc were seeded to generate spheroids, but at 48 hpi PBMCs were added (E:T = 10:1). Spheroid co-cultures were imaged and verified for EGFP expression using an EVOS fluorescent microscope (ThermoFisher Scientific). At indicated times, supernatants were collected to detect any NLuc released upon cancer cell death into the media. Native Coelenterazine substrate (CTZ, 303-500; NanoLight Technology) was added to salt buffer (5 µl of CTZ per ml of Renilla salts) to treat samples and results were obtained in solid white-bottom plates (TC-treated microplates, 3917; Corning) using a BioTek Microplate Reader, as previously described (63).

### Human xenograft and syngeneic murine model studies

Animal cohorts were randomized following tumour implantation before initiation of the treatment plan. Cells for *in vivo* studies were washed 2x with PBS on ice, strained (0.45 µm filter), counted by ViCell, and resuspended in PBS. Mouse weights were recorded 3x per week, and tumour size was measured 3x per week using calipers and tumour volume was calculated using a modified ellipsoidal formula; tumour volume=[(width^2^ x length)/2], where width is the smallest dimension. All subcutaneous tumours were injected upon measuring 62.5-108 mm^3^ at the indicated times. Mice were euthanized when tumour volumes reached above 1500 mm^3^ and according to the institutional guidelines for animal care. Mice were considered cured if mice survived and no tumours were present at day 100, and at this time, mice were rechallenged by subcutaneous bilateral injection of MC38_WT_ or MC38_CEA_ cells with the same number of cells (cell type indicated) from initial injections and monitored for 3 weeks.

In 8-week-old athymic nude mice (Charles River Laboratories; MA, USA), HT-29 cells (2E6) were injected subcutaneously in the right flank of the mice. Treatments of intratumoural injections of virus (3x 1E7 in 100 µl) and human PBMCs (1x or 2x 1E7 in 50 µl) were administered at indicated timepoints. In parallel, other mice were treated and endpointed to collect tumours for experiments (fixed in 10% neutral-buffered formalin for IHC or enzymatically dissociated for flow cytometry).

In 8-week-old immunocompetent C57BL/6J (The Jackson Laboratory; ME, USA), MC38_WT_ or MC38_CEA_ cells with Matrigel (5E5 cells in 50 µl of PBS combined with 50 µl Matrigel, 356231; Corning) were injected subcutaneously in the right flank of the mice. Treatments of intratumoural injections of virus (3x 1E7 in 100 µl) were administered at indicated timepoints. For the bilateral model, C57BL/6J mice were injected subcutaneously with MC38_CEA_ cells with Matrigel simultaneously in the left and right flank of the mice, but only the left tumour was treated with intratumoural injections of virus (3x 1E7 in 100 µl) at indicated timepoints. In parallel, other mice were treated and endpointed to collect tumours, which were enzymatically dissociated for flow cytometry, or for cytokine assessment according to manufacturer instructions (Proteome Profiler Mouse Cytokine Array Kit Panel A, ARY006, R&D Systems, MN, USA), along with serum in gel tubes (microcuvette 200 serum gel, 20.1291; Starstedt, Nümbrecht, Germany) separated by centrifugation at 15 000 g for 5 min at 20°C. Cytokine assay was quantified by densitometry. For *ex vivo* HiBiT detection from frozen and mechanically dissociated tumours, passive lysis buffer, LgBiT and Nanoglo substrate were used following manufacturer instructions (Nano-Glo HiBiT Extracellular Detection System, N2421; Promega).

For the peritoneal carcinomatosis model, MC38_CEA_ cells without Matrigel (5E5 cells in 100 µl of PBS) were injected intraperitoneally into the abdomen of human-CTLA4 C57BL/6N mice (C57BL/6-*CTLA4^tm1(CTLA4)Bcgen^*/Bcgen, 110011; Biocytogen, Beijing, China). Treatments of intraperitoneal injections of virus (4x 1E8 in 100 µl) with or without Anti-CTLA4 (SIM0004; Bio X Cell, NH, USA) or IgG control were administered at indicated timepoints. For the lung metastasis model, MC38_CEA_ cells without Matrigel (1E6 cells in 100 µl of PBS) were injected intravenously into the tail vein of human-CTLA4 C57BL/6N mice. Treatments of intravenous injections of virus (2x 1E8 in 100 µl) were administered at indicated timepoints before lungs were harvested. At day 15, lungs were injected intratracheally with black India ink. Once removed from the mice, lungs were rinsed with water and then fixed in Fekete’s solution (100 ml formalin, 700 ml ethanol, 50 ml glacial acetic acid, 150 ml distilled water).

### IHC staining

For IHC, an anti-vaccinia (CDRD, R1347-A056) antibody was used at a concentration of 12.5 µg/ml, or cleaved-caspase 3 antibody as previously described (64). Briefly, tissues were deparaffinized and hydrated (5 min each in xylene and alcohol). Antigen retrieval was performed in citrate buffer (pH 6.0, Vector H-3301) and endogenous peroxidase activity was quenched in 3% H_2_O_2_. Slides were incubated with primary antibody overnight at 4°C. Slides were incubated with secondary antibody (goat anti-rabbit) for 30 min at room temperature. After three washes in PBS slides were incubated with ABC solution (avidin biotin complex, Vector PK-6101) for 30 min at room temperature. After 3 washes in PBS slides were incubated with DAB (Vector, SK-4100). Slides were counterstained in hematoxylin for seconds and dehydrated through alcohol and xylene. Mounting media was used to add slides to were coverslips.

### Statistical analyses

Statistical analyses were performed using Prism 9 (GraphPad). Quantitative data are reported as mean ± SEM as indicated in the figure legends. Statistical analyses were performed on raw data by Student’s t test to compare two independent conditions, one-way ANOVA to compare three conditions or more, two-way ANOVA with Tukey’s correction (unless stated for Sidak’s correction) to compare groups influenced by two variables, and the Kaplan–Meier method followed by log-rank test for *in vivo* survival analyses. The statistical significance of all P values are: not significant (ns), P > 0.05, *P < 0.05, **P < 0.01, ***P < 0.001 and ****P < 0.0001. Differences between experimental groups were considered significant at P < 0.05. Exact P values are provided in the text or figure legends as indicated.

## Data availability statement

The original contributions presented in the study are included in the article/**Supplementary Material**. Further inquiries can be directed to the corresponding author.

## Ethics statement

Our research complies with all relevant ethical regulations at OHRI and the University of Ottawa (biohazardous material use certificate GC317-125-12). All colorectal tumour samples were obtained from cancer patients through the Global Tissue Consenting committee at the OHRI. The patients/participants provided their written informed consent to participate in this study. All animal studies were reviewed and approved by the institutional animal care committee of the University of Ottawa (Protocol ID: MEe-2258) and carried out following guidelines of the National Institutes of Health and the Canadian Council on animal care.

## Author contributions

MC, ZT, TJ, SB, OK, SV, EL, BH, BA, TA, conducted *in vitro* and *ex vivo* experiments. MC, ZT, TJ, JP, and AJ performed *in vivo* mouse studies. MC, ZT, TJ, NA, and SK completed and analyzed flow cytometry experiments. MC, ZT, SB, XH, RM, MH, LP, JD, AP, and SN engineered and manufactured viruses. MC, ZT, TJ, SB, LP and JB provided conceptualization, formal analyses and wrote the manuscript. MC, ZT, TJ, DF, MB, SM, RA, CI, JD and JB supervised and contributed to design of the studies. JB, CI, MC, LP provided funding acquisition. All authors contributed to the article and approved the submitted version.

## Funding

This work was funded by the Canadian Cancer Society Impact (#706162) grant to JB and CI and co-applicants MC and LP, as well as grants to JB and CI from the Canadian Institutes of Health Research (#377104) and generous support from the Ontario Institute for Cancer Research, the Ottawa Regional Cancer Foundation and the Ottawa hospital foundation. JD is supported by the CIHR New Investigator Award - Infection and Immunity (INI-147824). JD and JB hold grants from the Terry Fox Research Institute (TFF 122868) and the Canadian Cancer Society supported by the Lotte and John Hecht Memorial Foundation (703014). MC, ZT, TJ, SB, and TA received funding support from CanPRIME/Mitacs fellowships. MC is funded by the Taggart-Parkes Fellowship. ZT is funded by an NSERC CGS-D3 and Ontario Graduate Scholarship. SV is funded by a CIHR Master’s Scholarship. LP and TA were funded by CIHR postdoctoral awards. AP was funded by the Lebovic Fellowship Funding.

## Acknowledgments

We would like to thank other members of the Bell, Diallo, Ilkow, and Auer laboratories as well as colleagues at the National Research Council for feedback on this project. We thank the personnel of the Flow Cytometry Core Facility, Histology Core Facility, and Animal Care and Veterinary Services of the Faculty of Medicine at the University of Ottawa for their support. Graphical illustrations were designed by Jen Power, Scientific Graphic Designer [https://www.jenpower.ca]. The graphical illustration shown in **Supplementary Figure 1B** was made using BioRender (License agreement number: WF23CQBING).

## Conflict of interest

We declare that JB has an interest in Turnstone Biologics, which develops the oncolytic vaccinia virus as an OV platform. LP, MH, JD, AP, CB, DS and MB have worked for Turnstone Biologics. JB, CB, DS and MB are shareholders in Turnstone Biologics.

The remaining authors declare that the research was conducted in the absence of any commercial or financial relationships that could be construed as a potential conflict of interest.

## Publisher’s note

All claims expressed in this article are solely those of the authors and do not necessarily represent those of their affiliated organizations, or those of the publisher, the editors and the reviewers. Any product that may be evaluated in this article, or claim that may be made by its manufacturer, is not guaranteed or endorsed by the publisher.

## Supplementary material

Supplementary Figure 1 Generation of TCEs targeting CRC cells. **(A)** TCEs were designed with a scFv targeting CEA with high affinity was derived from MFE-23, a monoclonal antibody used in patients with colorectal cancer tumours (50); whereas the scFv that binds murine or human CD3ε was derived from well-characterized OKT3 or 145-2C11 antibodies, respectively. An upstream Ig Kappa leader sequence promotes secretion of the TCE. (**B)** A pipeline for validation of our TCE constructs (independent of oncolytic viruses). TCE constructs can be transfected into HEK293T cells, which will secrete TCEs in media that can be collect and concentrated. His-tagged TCEs can then be quantified by a His ELISA, and frozen at -80°C for long-term use. TCEs can be used for *in vitro* studies (e.g. immunoblotting or co-cultures) or *in vivo* studies. (**C)** We developed a TCE binding assay, whereby we can use an Alexa Fluor 647-conjugated His antibody to quantify TCE attachment to cell-surface CEA on cancer cells. Cells were placed on ice and treated with TCEs for 1 h prior to washing away excess unbound TCEs and quantifying the amount of TCEs bound to the cell surface of live cells. (**D)** By TCE binding assay, αCEA TCE specifically binds to CRC cells (HT-29, COLO 205; murine MC38 cells expressing human CEA/MC38_CEA_), pancreatic cancer cells (BxPC-3), breast cancer cells (MCF7) and lung cancer cells (A549), but not CEA-negative cells including glioblastoma U87MG and MC38_WT_. Results show relative MFI ± SEM; Two-way ANOVA. E) Whole cell lysates from indicated cancer cell lines were collected and immunoblotted for CEA and β-actin as a loading control.

Supplementary Figure 2 Validation of TCEs targeting CRC cells. **(A)** HCT 116 cells were co-cultured with human PBMCs (E:T = 5:1) and αCEA TCE (αCEA:hCD3) or αCEA CTRL (αCEA:mCD3) at indicated concentrations. Resazurin assay was performed at 72 h to determine cancer cell viability after TCE treatment. We did not detect any significant change in cell viability of HCT 116 CEA-negative CRC cells in co-cultures. Results show relative % ± SEM. **(B)** BxPC-3 cells were co-cultured with human PBMCs (E:T = 5:1) and αCEA TCE (αCEA:hCD3) or αCEA CTRL (αCEA:mCD3) at indicated concentrations. Resazurin assay was performed at 72 h to determine cancer cell viability after TCE treatment. Increasing amounts of αCEA TCE, but not αCEA CTRL, promote a greater reduction in cell viability of BxPC-3 pancreatic cancer cells. The αCEA CTRL did not cross-react with the opposite species of CD3ε T cell population, as no effect was observed with this negative control in co-cultures, as predicted. Results show relative % ± SEM. **(C)** MIA PaCa-2 cells were co-cultured with human PBMCs (E:T = 5:1) and αCEA TCE (αCEA:hCD3) or αCEA CTRL (αCEA:mCD3) at indicated concentrations. Resazurin assay was performed at 72 h to determine cancer cell viability after TCE treatment. We did not detect any significant change in cell viability of MIA PaCa-2 CEA-negative CRC cells in co-cultures. Results show relative % ± SEM. **(D)** PBMCs were cultured alone or cultured with HT-29 cells (E:T = 5:1) with addition of αCEA TCE or αCEA CTRL (1 µg/ml). CD69 expression measured using flow cytometry after 24h of co-culture. A significant increase in CD69 levels of both CD4-positive and CD8-positive populations of CD3-positive T cells was detected in co-culture models with PBMCs in the presence of HT-29 cells and αCEA TCE. Gated for live, CD3+, CD4+ and CD8+ cells. Results show frequency of CD69+ cells as % ± SEM. **(E)**Whole cell lysates were collected and immunoblotted for CEA and β-actin as a loading control. Increasing doses of the current standard of care drug for colorectal cancer, 5-Fluorouracil (5-FU), increases CEA levels in COLO 205 and SW620 colorectal cancer cell lines that initially express low levels of CEA. We did not detect an increase in HCT 116 and HCT15 CRC cell lines that do not express basal levels of CEA, and we did not detect a noticeable increase in HT-29 cells that express an abundant level of CEA at basal levels. (**F)** By TCE binding assay, 5-FU enhanced αCEA TCE binding to the surface of COLO 205 and SW620 cell lines. 5-FU did not increase αCEA TCE binding to CEA-negative cell lines HCT 116 and HCT15, or to HT-29 cells which express the highest levels of CEA in the absence and presence of 5-FU and which bind most robustly to αCEA TCE.

Supplementary Figure 3 TCE limitations identified by HiBiT TCE-tagging technology. **(A)** TCEs were designed by fusing to a HiBiT peptide for bioluminescent quantitation. The 11-amino-acid HiBiT tag was added to either the αCD3 scFv (HiBiT_A_) or the αCEA scFv (HiBiT_B_) of the TCE. **(B)** HEK293T cells were transiently transfected with the two individual constructs and lysates and supernatants were collected at 48 h post-transfection. Both TCE versions were detected in lysates by immunoblotting and probing with Large BiT (LgBiT), the complementary polypeptide that binds HiBiT with high affinity. **(C)** The luminescent signal was quantified from the supernatant of cells transfected with the constructs. We detected higher levels of luminescence with HiBiT_B_ in a dynamic range that can be used to measure TCEs *in vivo*. **(D)** We concentrated αCEA TCE-HiBiT_B_ to treat mice bearing colorectal tumours (MC38_WT_). We injected a single dose of 10 μg of TCE intratumourally and quantified the presence of a luminescent signal *ex vivo* from collected mouse serum or dissociated tumours at different time points after treatment. We show that the luminescent signal decreases over time in tumours, suggesting that the TCE has a short half-life, consist with other studies. We detect TCE presence in the serum which also decreases overtime, indicating that the tumour is leaky and may not retain all the TCE in the TME.

Supplementary Figure 4 Flow cytometry gating strategy. **(A)** Gating strategy for T and NK immune cell populations.

Supplementary Figure 5 Strategically combining oncolytic vaccinia virus and TCEs targeting CEA. **(A)** CEA depletion by siRNA does not negatively impact oncolytic vaccinia virus replication, compared to non-targeting control (NTC) knockdown. **(B)** Individual tumour volumes show improved efficacy in decreasing tumour burden with combination treatment of αCEA TCE (αCEA:mCD3) and VV-CTRL, compared to αCEA TCE (αCEA:mCD3) and PBS, or PBS alone. **(C)** Plasmids with appropriate homology arms were designed and used to insert transgenes into the B14R/3p locus of the Copenhagen vaccinia virus genome, under control of a vaccinia late promoter. Infection with oncolytic vaccinia virus (Cop 3p-5p-; no fluorescent tag) and transfection of the plasmid allowed for recombination to occur. Fluorescent plaques were selected, purified and sequenced to generate 3 viruses expressing: eGFP only (VV-CTRL); αCEA:mCD3 + eGFP + (VV-αCEA:mCD3), as well as αCEA:hCD3 + eGFP (VV-αCEA:hCD3). **(D)** Visualization of eGFP upon infection of HEK293T cells with viruses at indicated timepoints and MOIs. Scale bar = 1000 µm. **(E)** Detection of His-tagged TCEs secreted from infected HEK293T cells at different timepoints and MOIs by immunoblotting the collected supernatants and probing with a His antibody.

Supplementary Figure 6 Verification of BEVir infection of CRC cell lines and oncolytic vaccinia-virus-mediated TCE production. **(A)** Visualization of eGFP upon infection of HT-29 cells with viruses at MOI 0.1 or MOI 1 at 48 hpi. Scale bar = 600 µm. **(B)** Visualization of eGFP upon infection of MC38_CEA_ cells with viruses at MOI 0.1 or MOI 1 at 48 hpi. Scale bar = 600 µm. **(C)** Detection of His-tagged TCEs in whole cell lysates and supernatants from HEK293T cells infected at MOI 1 at 48 hpi by immunoblotting and probing with a His antibody. Vaccinia was detected in the whole cell lysates of infected cells, and ponceau staining was used to see total protein and equal loading. **(D)** Detection of His-tagged TCEs in whole cell lysates and supernatants from MC38_CEA_ cells infected at MOI 1 at 48 hpi by immunoblotting and probing with a His antibody. Vaccinia was detected in the whole cell lysates of infected cells, and ponceau staining was used to see total protein and equal loading.

Supplementary Figure 7 Verification of oncolytic vaccinia virus replication and lytic activity in the absence of T cells. The viral titers (log_10_ pfu/ml) were determined for VV-CTRL, VV-αCEA:hCD3, and VV-αCEA:mCD3 at indicated times, following infection of **(A)** HT-29, **(C)** BxPC-3, **(E)** COLO 205, **(G)** MC38_CEA_, and **(I)** MC38_WT_ cell lines. The insertion of a TCE transgene did not interfere with viral replication. Viral lytic activity of VV-CTRL, VV-αCEA:hCD3, and VV-αCEA:mCD3 at indicated times, following infection of **(B)** HT-29, **(D)** BxPC-3, **(F)** COLO 205, **(H)** MC38_CEA_, and **(J)** MC38_WT_ cell lines. Cell viability did not differ between viruses in multiple human and murine cancer cell lines, in the absence of T cells. Resazurin assay was performed at indicated times to determine cancer cell viability after virus treatment. Results show relative % ± SEM.

Supplementary Figure 8 Bystander mediated effects of TCEs produced by infected cells. **(A)** To better visualize co-cultures, we generated HT-29 cells that express Azurite as a fluorescent marker, infected with VV-αCEA:hCD3, which generates eGFP-positive plaques, and PBMCs stained with a red CellTracker dye. **(B)** HEK293T cells were infected with VV-CTRL, VV-αCEA:hCD3, and VV-αCEA:mCD3 viruses, and supernatants were filtered (to remove vaccinia viruses) at 48 hpi. Supernatants containing TCEs were concentrated. HT-29 and COLO 205 cells were then treated with the collected supernatants, demonstrating a bystander T-cell effect on our J69 reporter line. We detected an increase in td-Tomato fluorescence intensity in conditions treated with filtered supernatants from VV-αCEA TCE (CEA:hCD3) but not other virus controls. Results show MFI ± SEM; Two-way ANOVA. **(C)** Co-cultures with CEA-negative MC38_WT_ cancer cells were infected 24 h after seeding at MOI 0.01. Inoculation medium was removed after 2 h and new medium was added containing murine effector cells (E:T = 5:1, or 0:1 as a negative control). Cell viability was assessed by resazurin assay and showed no decrease in cell viability, in the presence of murine effector cells. Results show relative % ± SEM. **(D)** Spheroids were imaged after TCE and VV-CTRL treatments, but the spheroids did not breakdown with αCEA TCE (CEA:hCD3) addition at different concentrations. Scale bar = 500 µm.

Supplementary Figure 9 Safety and efficacy of VV encoding TCE in a human xenograft model. **(A)** No significant differences were observed in weights of mice after injection with the different viruses and/or PBMCs. **(B)** No pox lesions on the tails and limbs of immunocompromised mice were detected, compared to mice injected with VV wildtype. **(C)** Mice bearing HT-29 tumours and co-injected with VV-αCEA TCE and PBMCs had a significant decrease in individual tumour volumes. **(C)** Schematic illustrating TCEs forcing the interaction between cancer cells and T cells, leading to release of granzyme B and perforin to activate caspases.

Supplementary Figure 10 Safety and efficacy of VV encoding TCE in immunocompetent mouse models. **(A)** Mice bearing MC38_CEA_ tumours treated with viruses showed decreased individual tumour volumes, compared to PBS controls. **(B)** No significant differences were observed in weights of mice after injection with the different viruses. **(C)** Mice bearing MC38_WT_ tumours showed decreased individual tumour volumes, compared to PBS controls. **(D)** All mice rechallenged with bilateral MC38_WT_ and MC38_CEA_ tumours had decreasing individual tumour volumes and rejected tumour engraftment compared to naïve mice controls.

Supplementary Figure 11 VV encoding TCE leads to abscopal effects and different cytokine profiles. **(A)** Mice bearing MC38_CEA_ bilateral tumours showed decreased individual tumour volumes, compared to PBS controls. In some mice, we observed the regression of both simultaneously engrafted bilateral MC38_CEA_ tumours. **(B)** A murine cytokine assay was performed using serum from mice with subcutaneous MC38_CEA_ tumours treated with VV-αCEA TCE, VV-CTRL or PBS. **(C–H)** Murine Cytokine Array blots. **(I)** Whole cell lysates were collected and immunoblotted for CEA and β-actin as a loading control. Human IFNγ treatment upregulates CEA levels on colorectal cancer cell lines.

Supplementary Figure 12 Combination viro-immunotherapy for peritoneal carcinomatosis and CRC metastases. **(A)** In a highly aggressive CRC model of peritoneal carcinomatosis, tumours grow upon the gut, but not other organs. **(B)** C57BL/6N mice that express human CTLA4 instead of murine CTLA4 on T cells and bear intraperitoneal MC38_CEA_ tumours, were injected intraperitoneally with 4 doses of VV-αCEA TCE (αCEA:mCD3) or VV-αCEA CTRL (αCEA:hCD3) viruses at 1E8 pfu, or PBS as control, at days 3, 4, 5 and 6. In addition, mice were co-injected intraperitoneally with αCTLA4 (1 mg/kg) or IgG control on days 4 and 6. **(C)** Antitumour efficacies of the combination treatments were assessed. Mice bearing MC38_CEA_ tumours treated with VV-αCEA TCE (αCEA:mCD3) and αCTLA4 showed significantly prolonged survival and some cures in this aggressive peritoneal carcinomatosis model. **(D)** Mice treated as indicated were generally endpointed before day 25, due to abdominal distension and respiratory distress. **(E)** An αFAP TCE was encoded into vaccinia virus (VV-αFAP TCE) to assess *in vivo* efficacy of the therapy. C57BL/6J mice bearing subcutaneous MC38_WT_ tumours were injected intratumourally with 3 doses of viruses at 1E7 pfu, or PBS as control, at days 6, 8 and 10 (similar to ). Antitumour efficacies of VV-CTRL, VV-αFAP TCE (CEA:mCD3) viruses were assessed in immunocompetent mice. Mice bearing MC38_WT_ tumours treated with virus showed improved survival with VV-αFAP TCE.
