# Supplementary material for: Oncolytic virus driven T-cell-based combination immunotherapy platform for colorectal cancer
Source: Front Immunol. 2022 Nov 3;13:1029269. doi: 10.3389/fimmu.2022.1029269 (PMC9670134; doi:10.3389/fimmu.2022.1029269)
Supplement: Supplementary Figure 1 — Generation of TCEs targeting CRC cells. (A) TCEs were designed with a scFv targeting CEA with high affinity was derived from MFE-23, a monoclonal antibody used in patients with colorectal cancer tumours (50); whereas the scFv that binds murine or human CD3ε was derived from well-characterized OKT3 or 145-2C11 antibodies, respectively. An upstream Ig Kappa leader sequence promotes secretion of the TCE. (B) A pipeline for validation of our TCE constructs (independent of oncolytic viruses). TCE constructs can be transfected into HEK293T cells, which will secrete TCEs in media that can be collect and concentrated. His-tagged TCEs can then be quantified by a His ELISA, and frozen at -80°C for long-term use. TCEs can be used for in vitro studies (e.g. immunoblotting or co-cultures) or in vivo studies. (C) We developed a TCE binding assay, whereby we can use an Alexa Fluor 647-conjugated His antibody to quantify TCE attachment to cell-surface CEA on cancer cells. Cells were placed on ice and treated with TCEs for 1 h prior to washing away excess unbound TCEs and quantifying the amount of TCEs bound to the cell surface of live cells. (D) By TCE binding assay, αCEA TCE specifically binds to CRC cells (HT-29, COLO 205; murine MC38 cells expressing human CEA/MC38CEA), pancreatic cancer cells (BxPC-3), breast cancer cells (MCF7) and lung cancer cells (A549), but not CEA-negative cells including glioblastoma U87MG and MC38WT. Results show relative MFI ± SEM; Two-way ANOVA. E) Whole cell lysates from indicated cancer cell lines were collected and immunoblotted for CEA and β-actin as a loading control. [file DataSheet_1.pdf]

Supplementary Figure 1

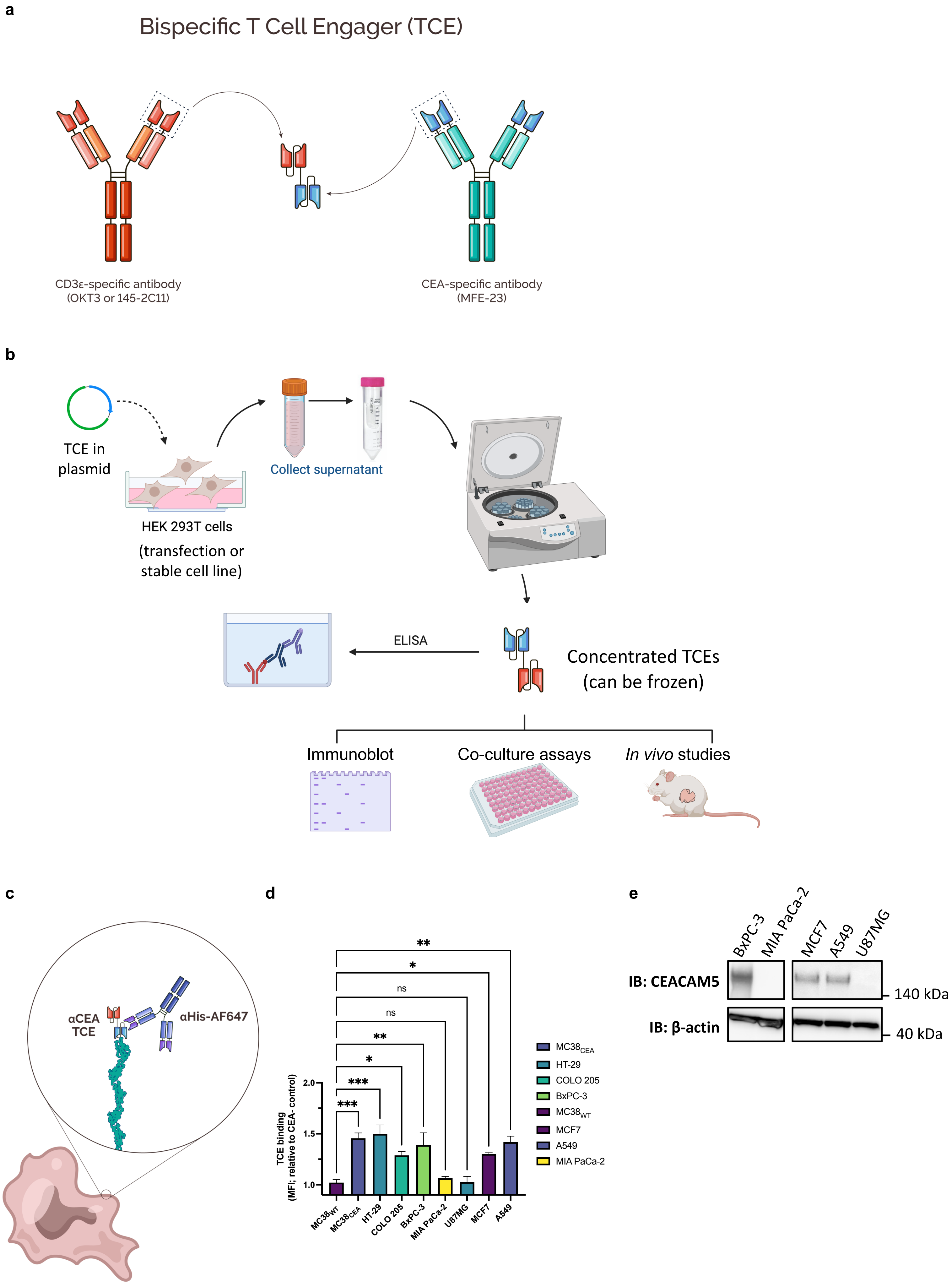

Supplementary Figure 2

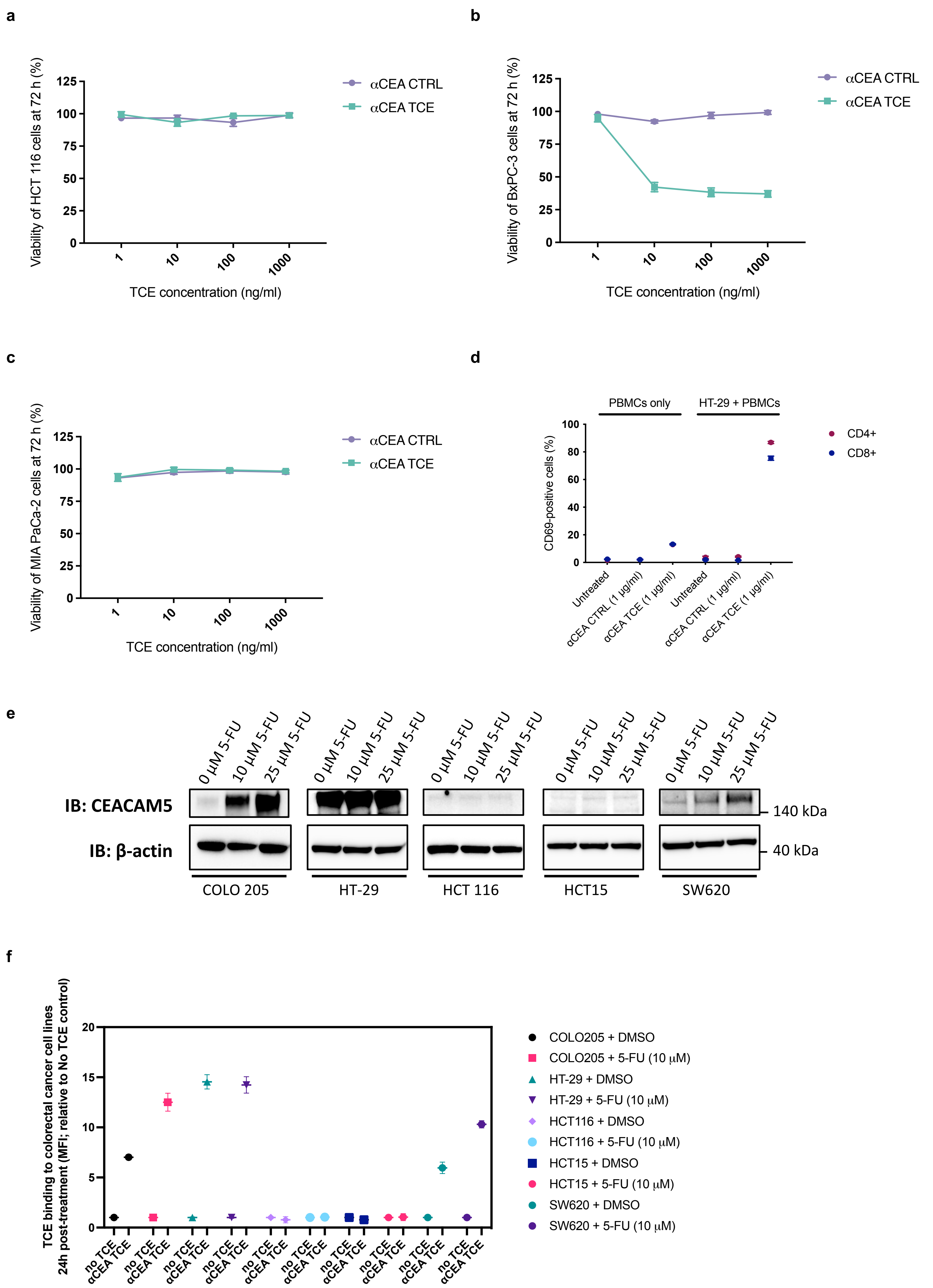

Supplementary Figure 3

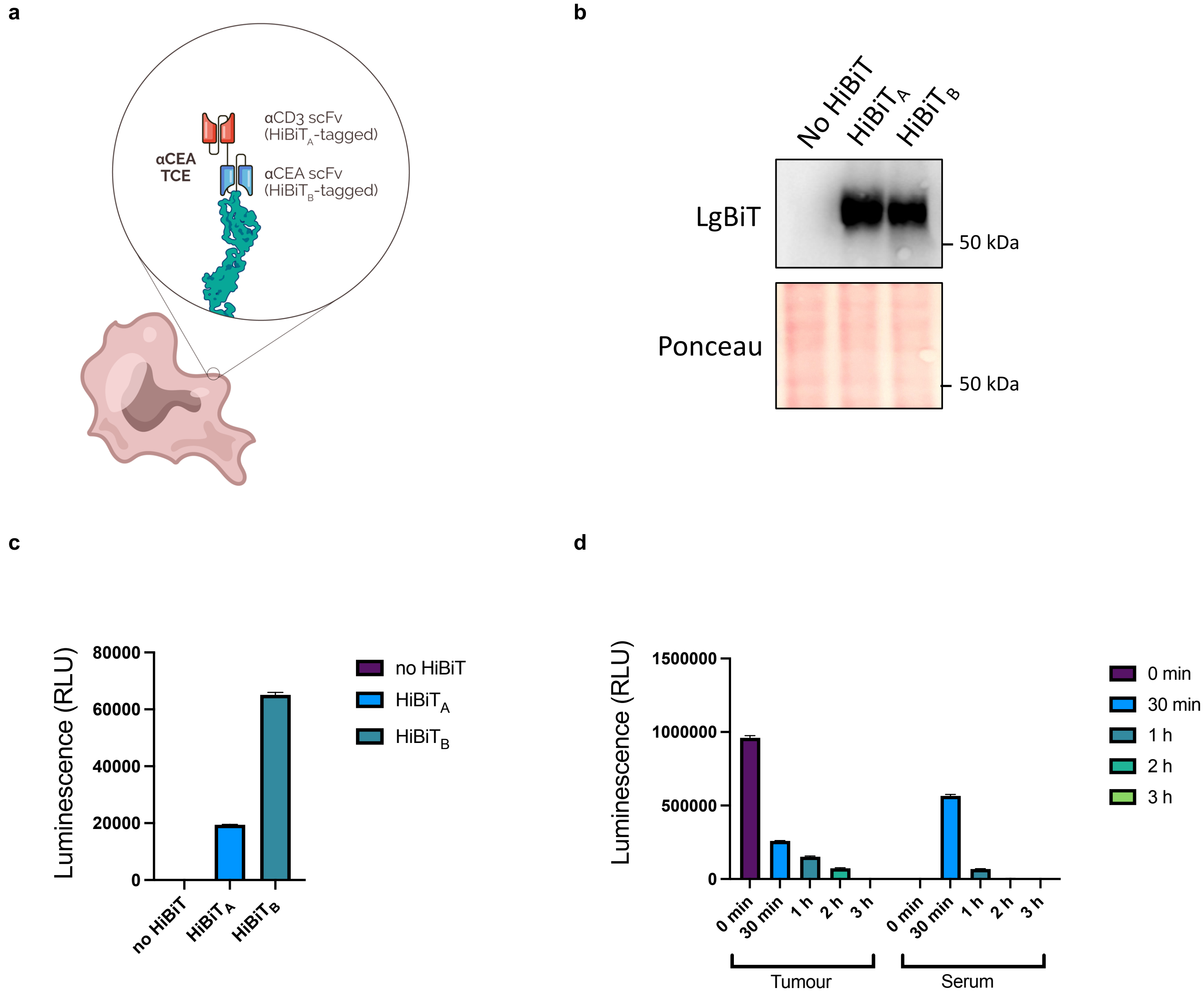

a

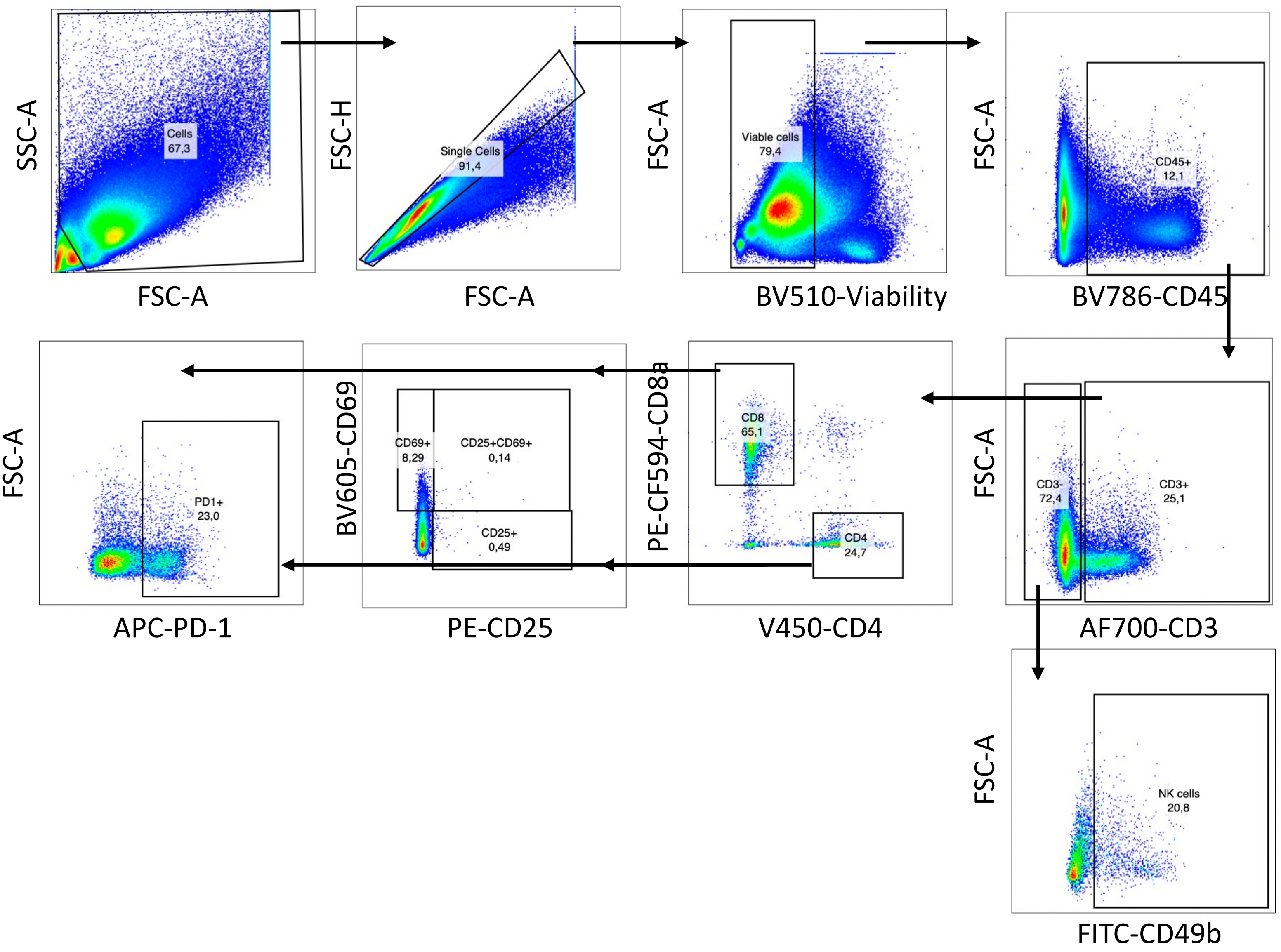
SSC-A
Cells
67,3
FSC-A
FSC-H
Single Cells
91,4
FSC-A
FSC-A
Viable cells
79,4
BV510-Viability
FSC-A
CD45+
12,1
BV786-CD45
FSC-A
CD3-
72,4
CD3+
25,1
AF700-CD3
PE-CF594-CD8a
CD8
65,1
CD4
24,7
V450-CD4
BV605-CD69
CD69+
8,29
CD25+CD69+
0,14
CD25+
0,49
PE-CD25
FSC-A
PD1+
23,0
APC-PD-1
FSC-A
NK cells
20,8
FITC-CD49b

Supplementary Figure 5

a

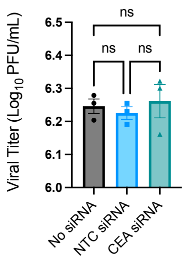

b

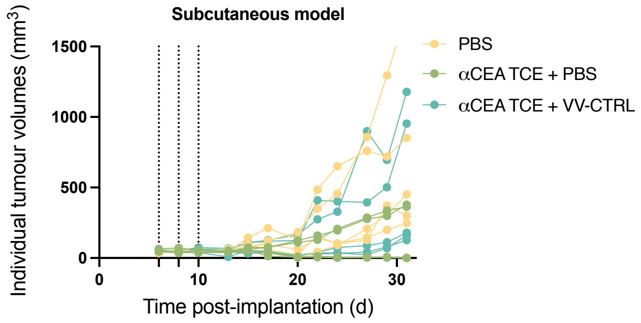

c

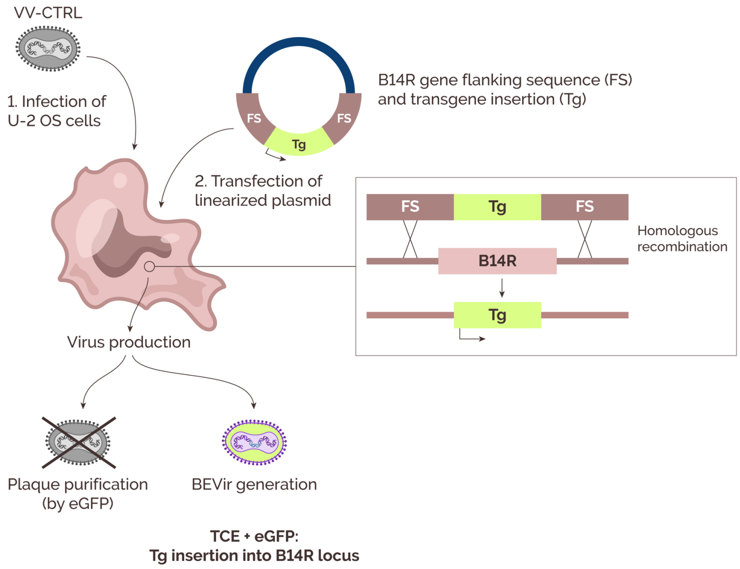

d

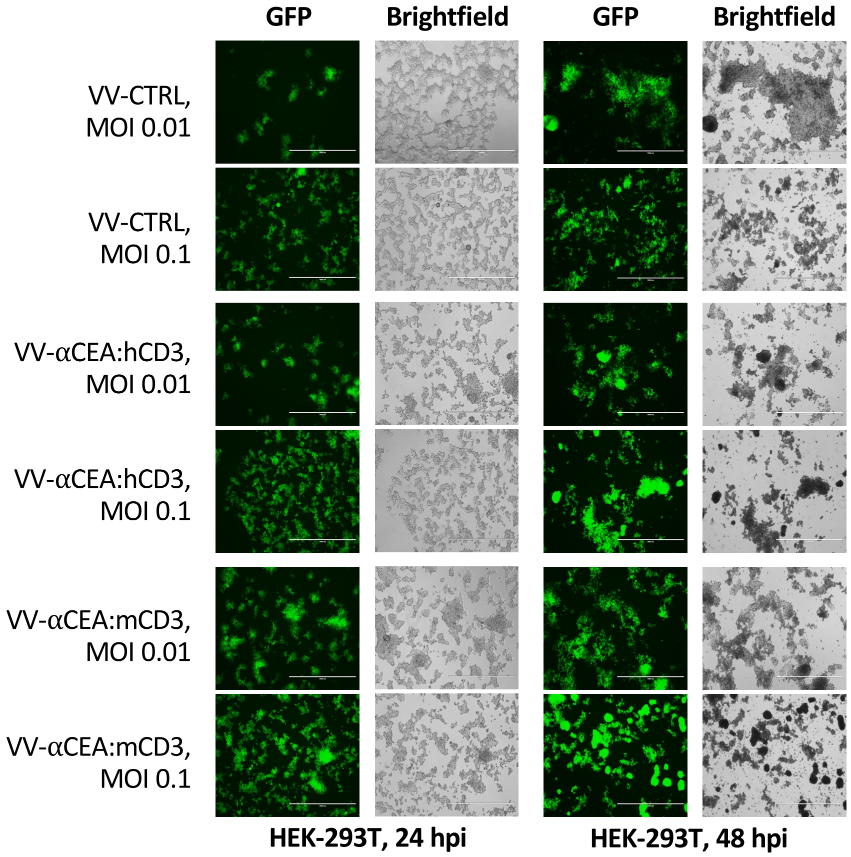

e

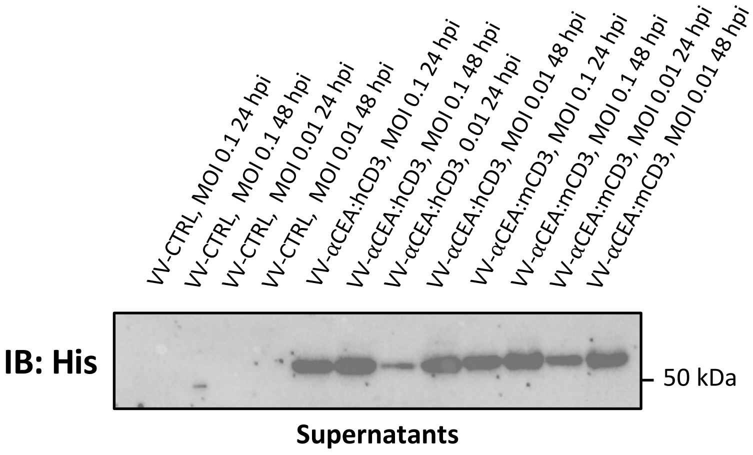

Supplementary Figure 6

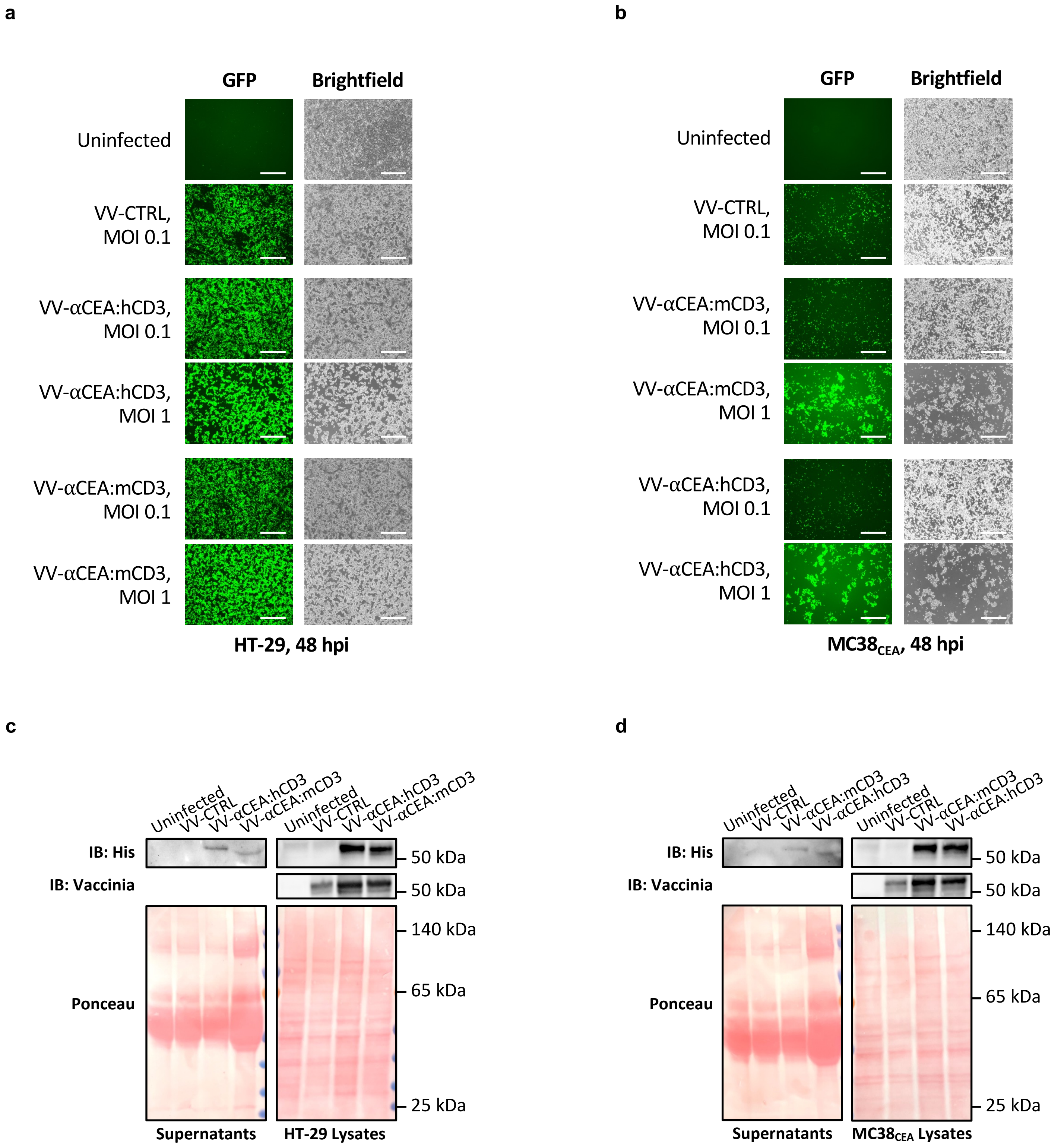

Supplementary Figure 7

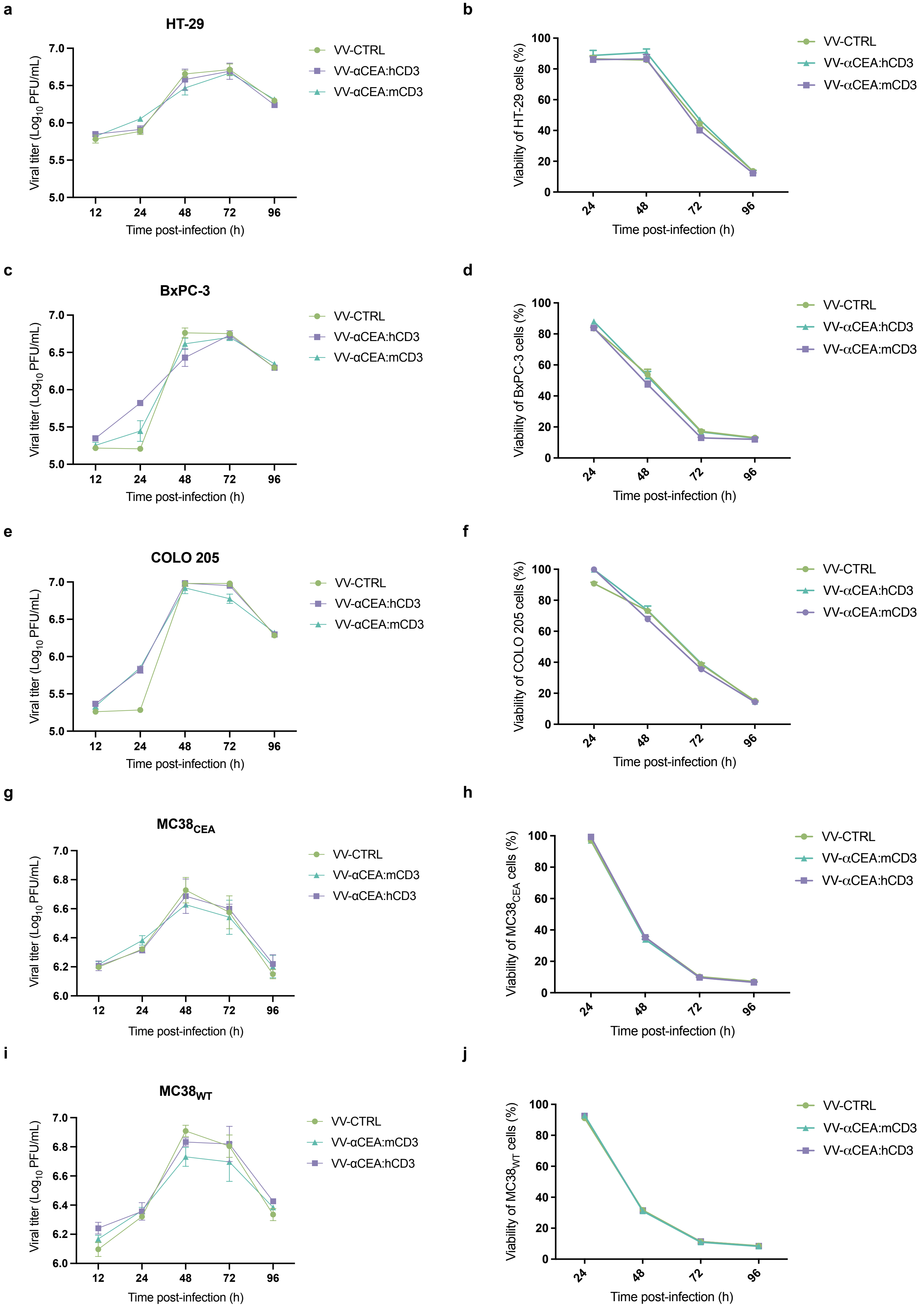

Supplementary Figure 8

a

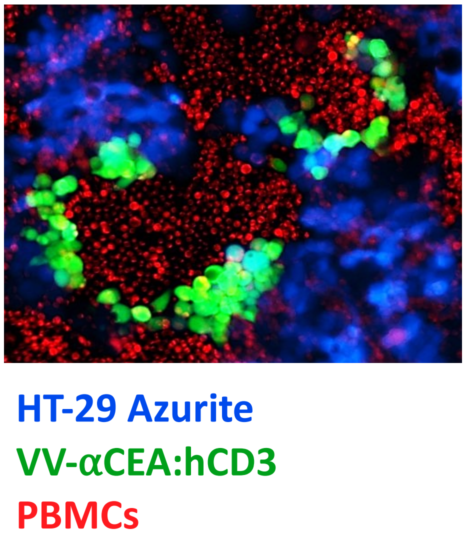

b

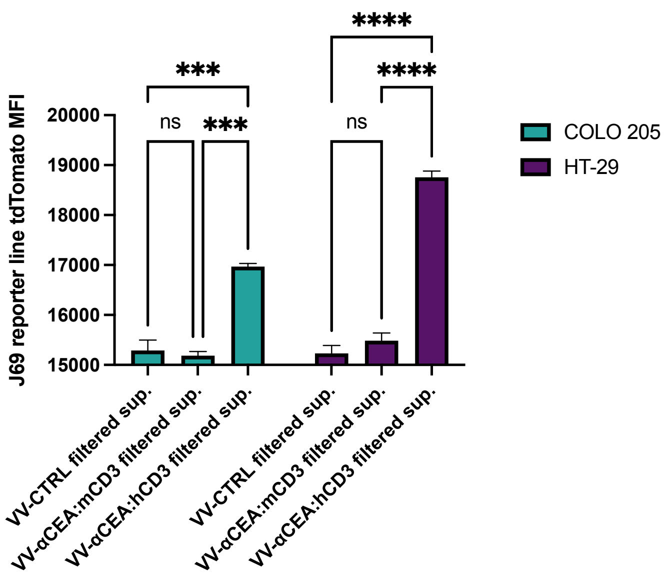

c

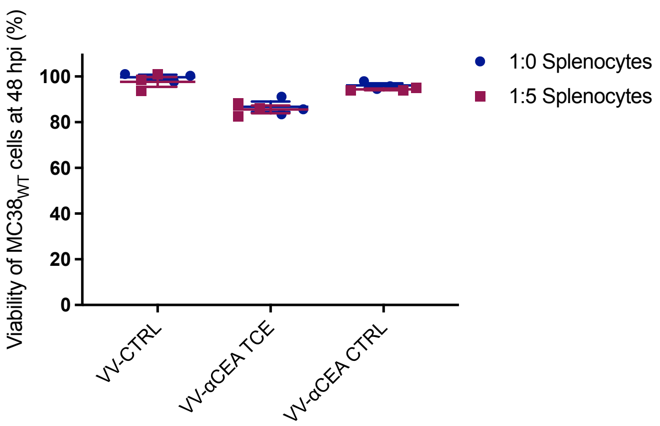

d

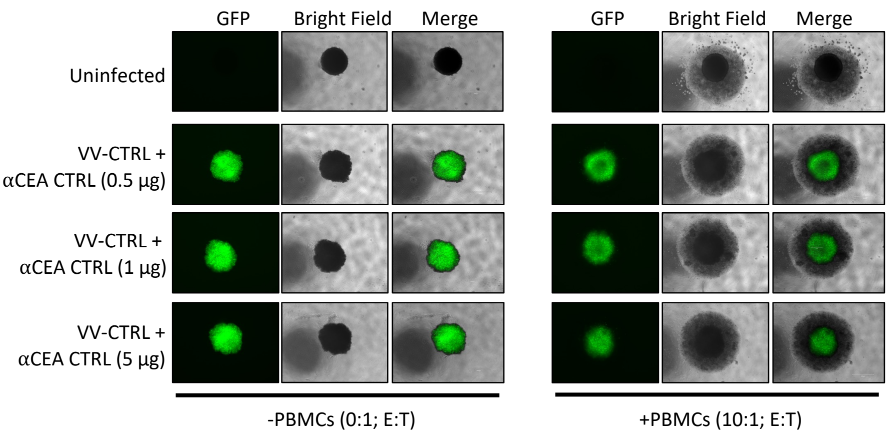

Supplementary Figure 9

a

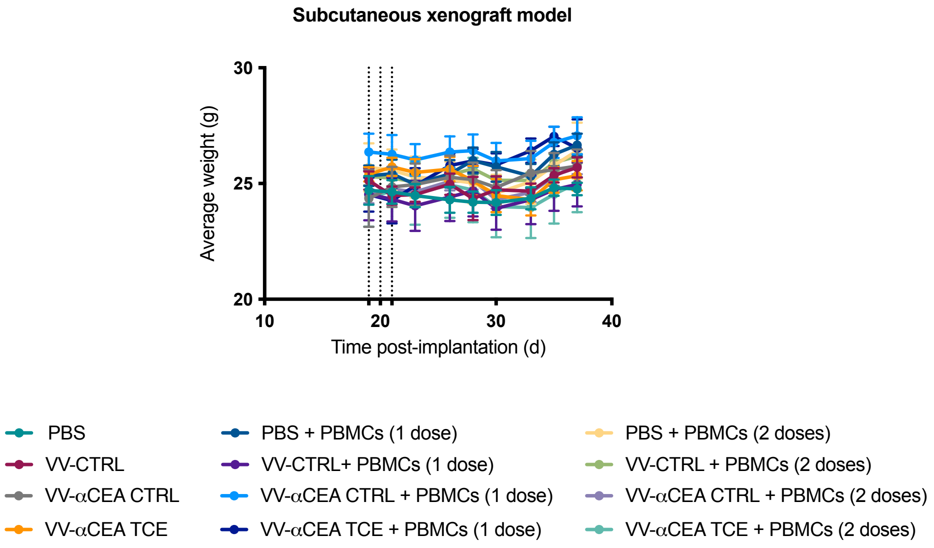

b

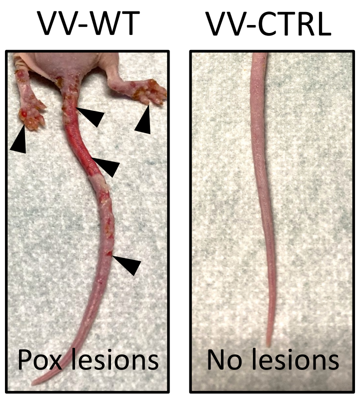

c

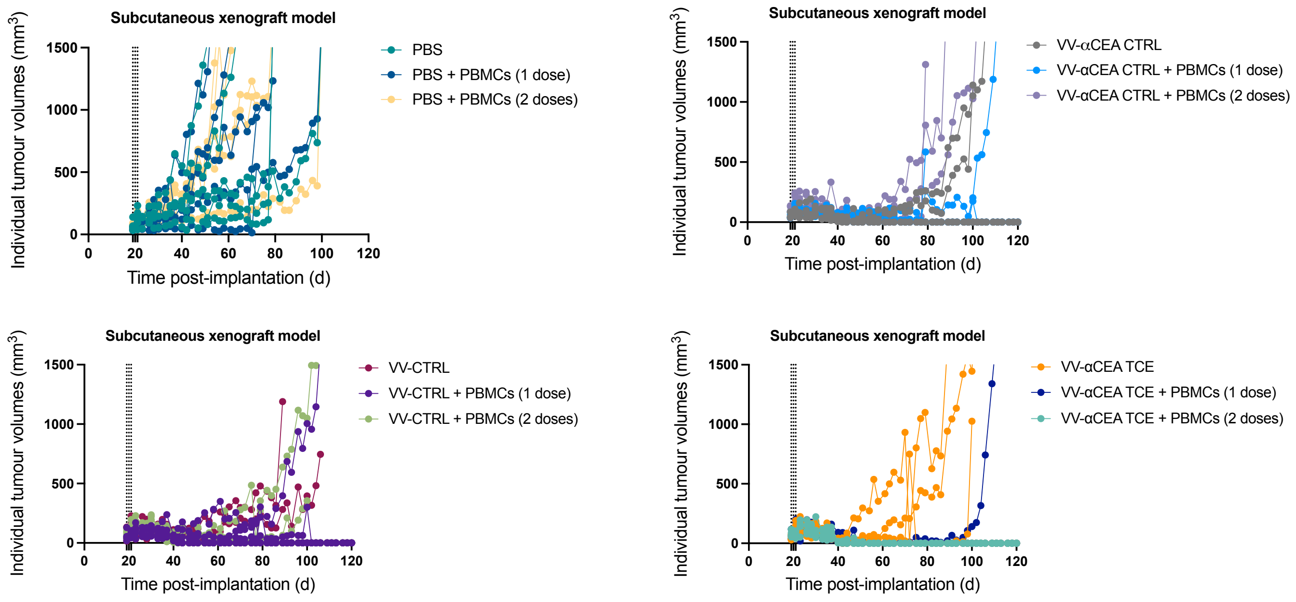

d

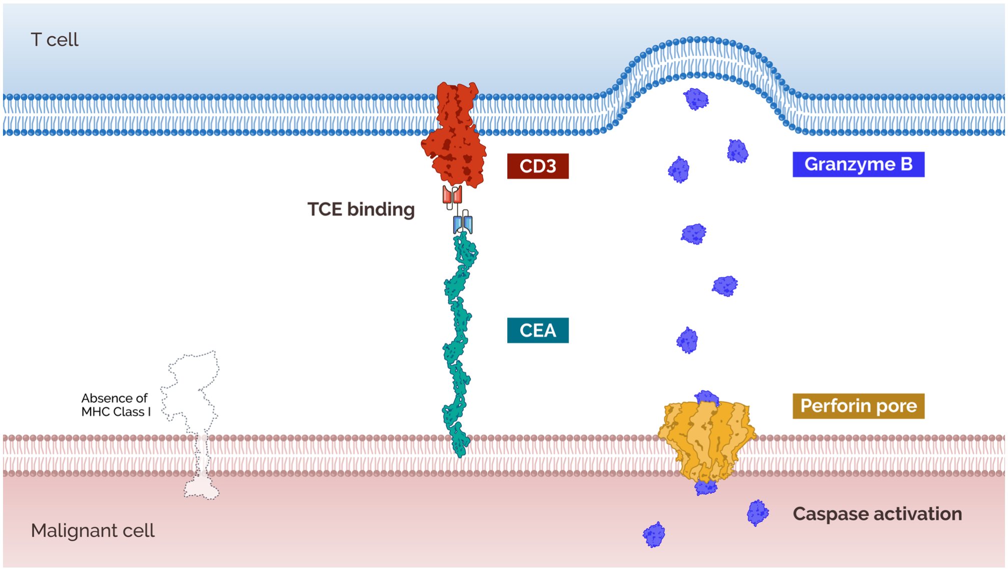

Supplementary Figure 10

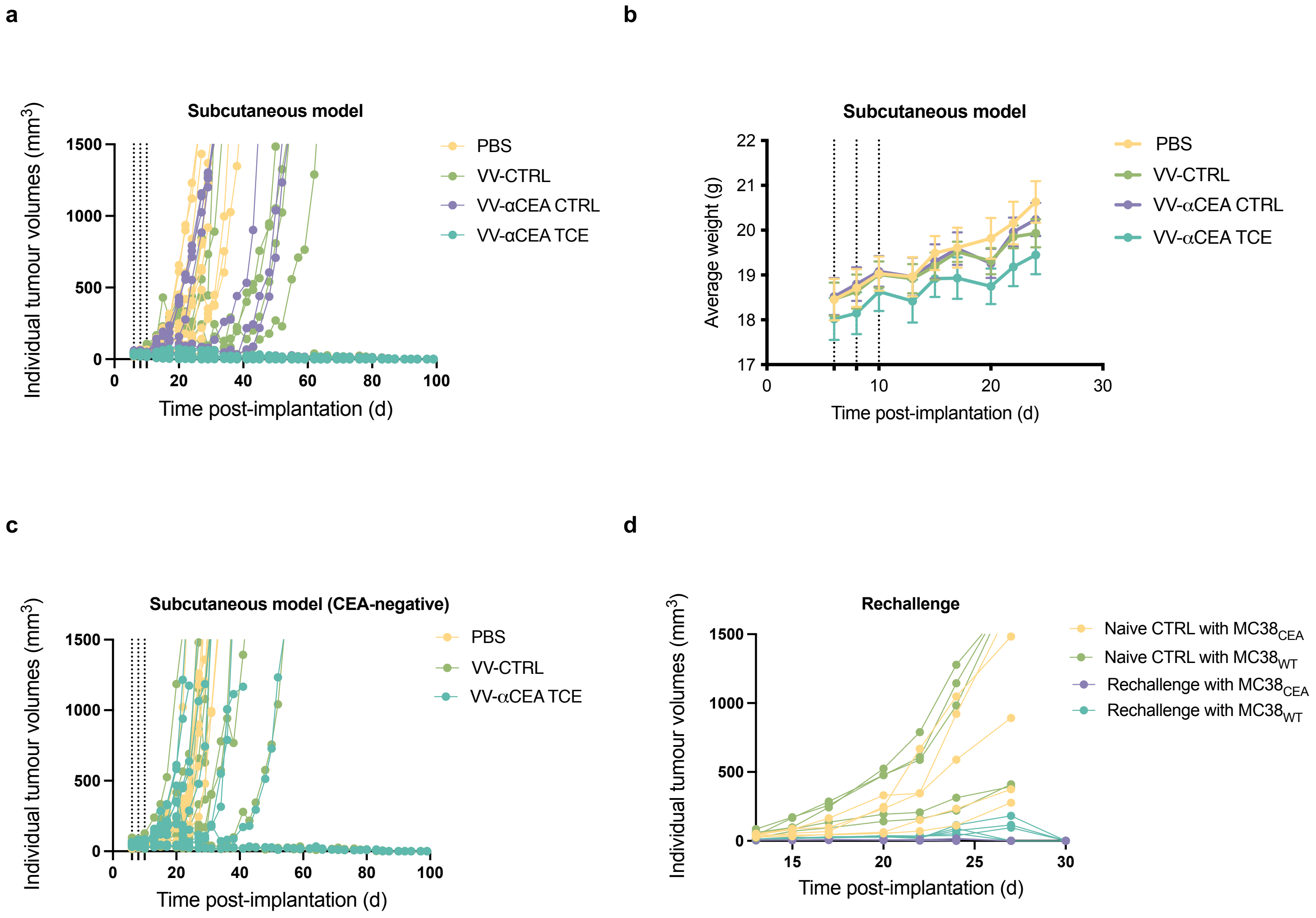

Supplementary Figure 11

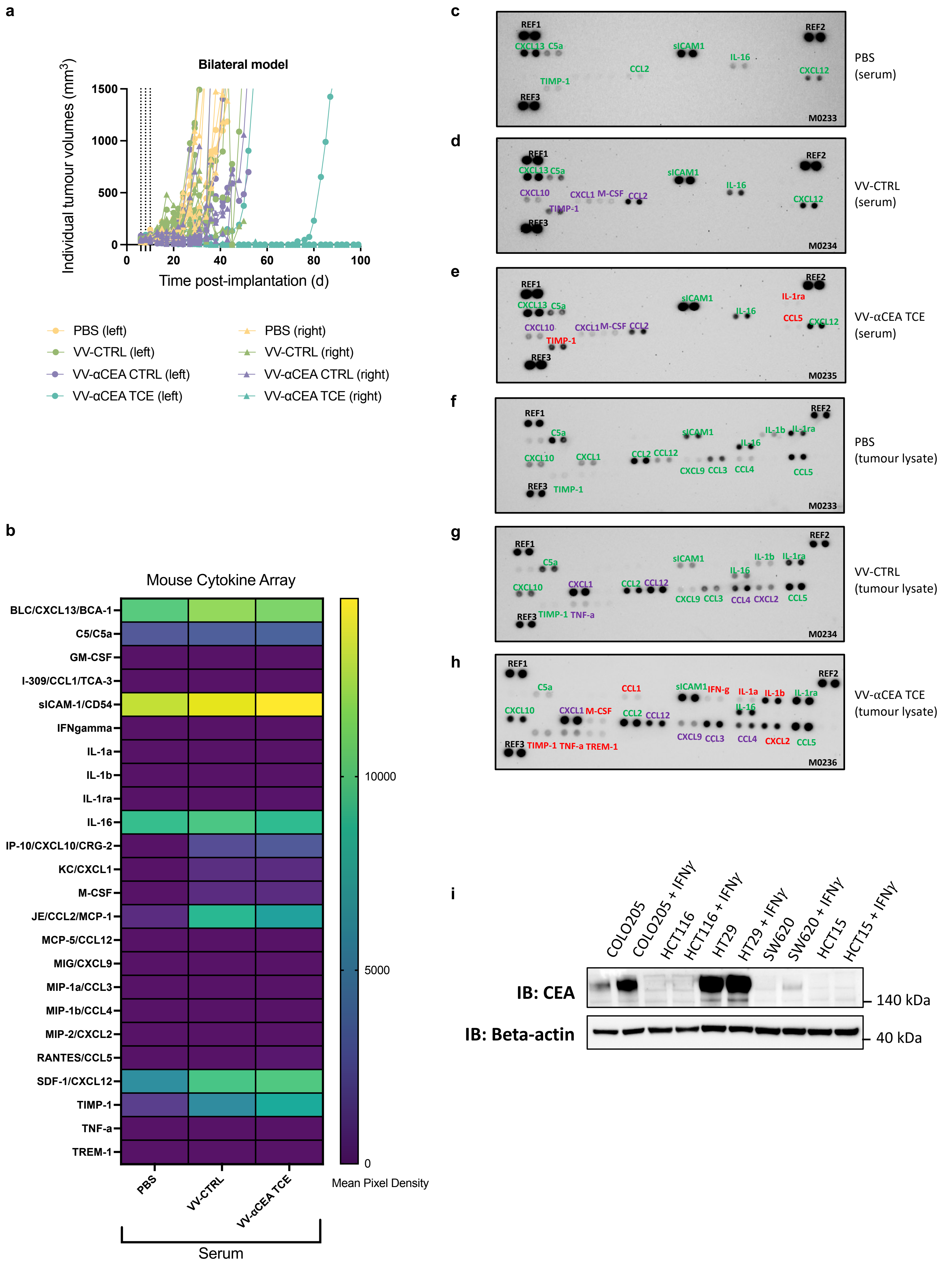

a

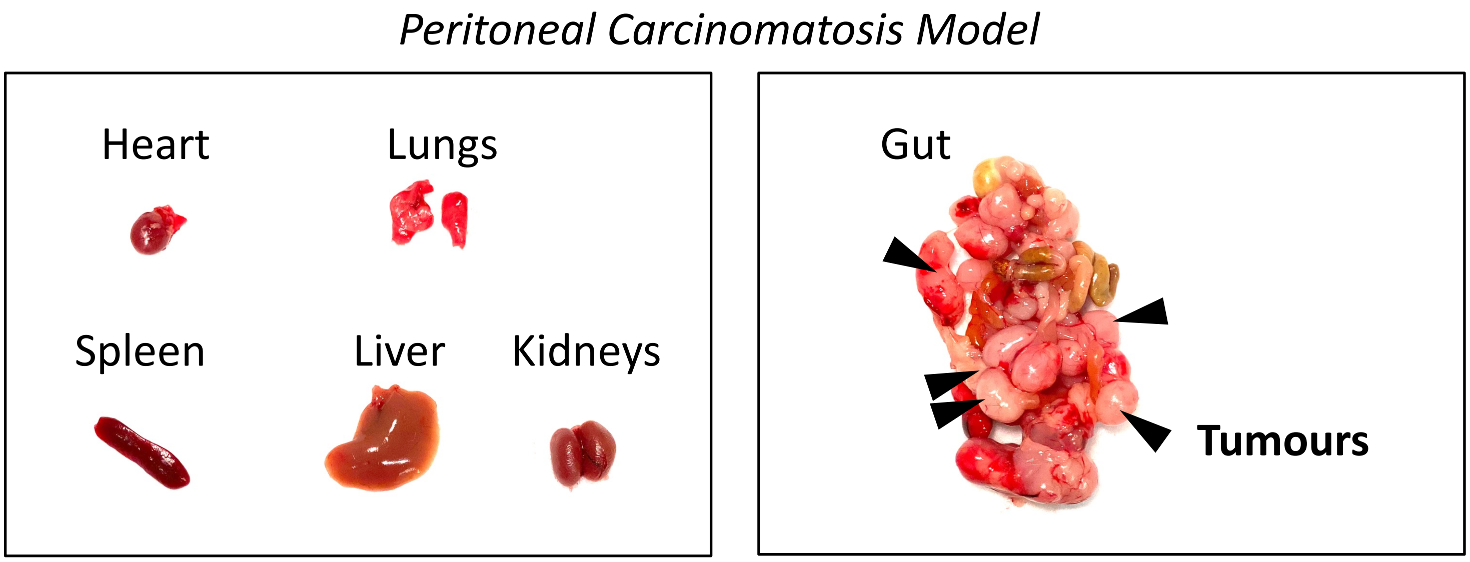
Peritoneal Carcinomatosis Model
Heart
Lungs
Spleen
Liver
Kidneys
Gut
Tumours

b

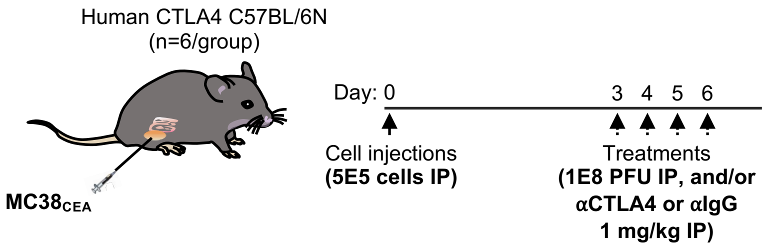
Human CTLA4 C57BL/6N
(n=6/group)
MC38CEA
Day: 0
3 4 5 6
Cell injections
(5E5 cells IP)
Treatments
(1E8 PFU IP, and/or
αCTLA4 or αIgG
1 mg/kg IP)

c

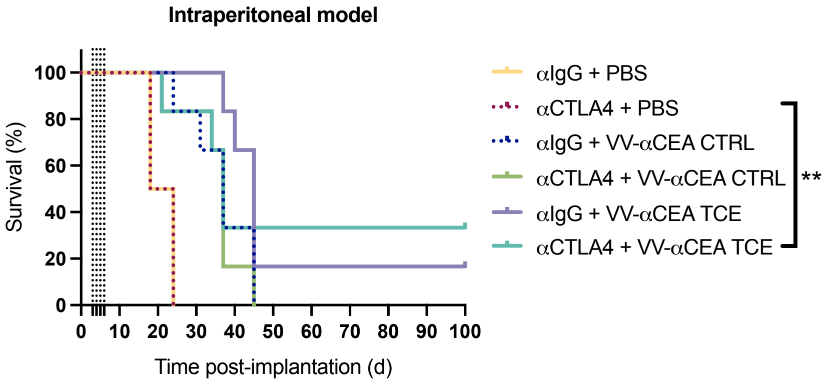
Intraperitoneal model
Survival (%)
Time post-implantation (d)
αIgG + PBS
αCTLA4 + PBS
αIgG + VV-αCEA CTRL
αCTLA4 + VV-αCEA CTRL
αIgG + VV-αCEA TCE
αCTLA4 + VV-αCEA TCE
**

d

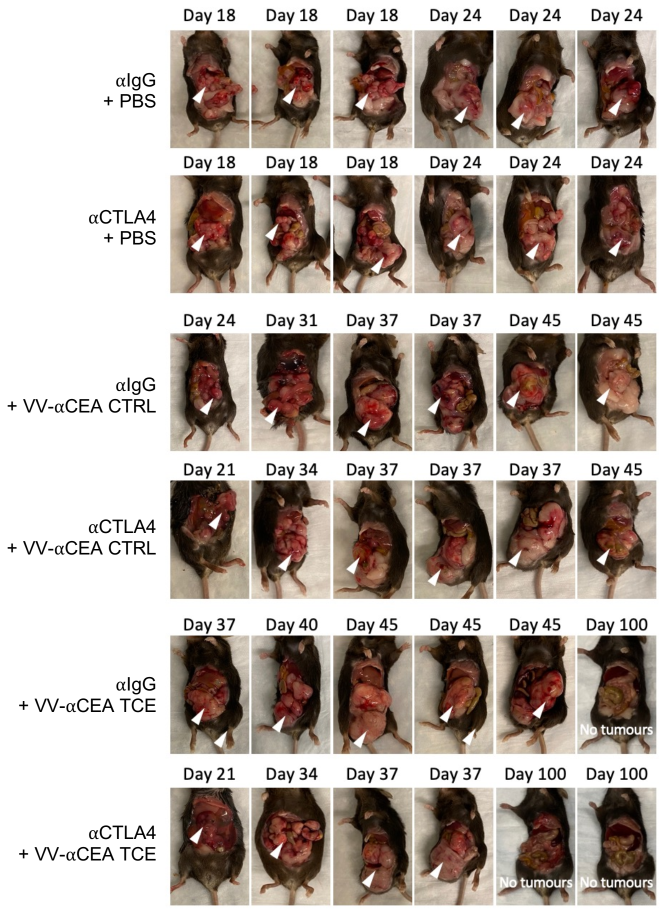
αIgG + PBS
Day 18 Day 18 Day 18 Day 24 Day 24 Day 24
αCTLA4 + PBS
Day 18 Day 18 Day 18 Day 24 Day 24 Day 24
αIgG + VV-αCEA CTRL
Day 24 Day 31 Day 37 Day 37 Day 45 Day 45
αCTLA4 + VV-αCEA CTRL
Day 21 Day 34 Day 37 Day 37 Day 37 Day 45
αIgG + VV-αCEA TCE
Day 37 Day 40 Day 45 Day 45 Day 45 Day 100
No tumours
αCTLA4 + VV-αCEA TCE
Day 21 Day 34 Day 37 Day 37 Day 100 Day 100
No tumours
No tumours

e

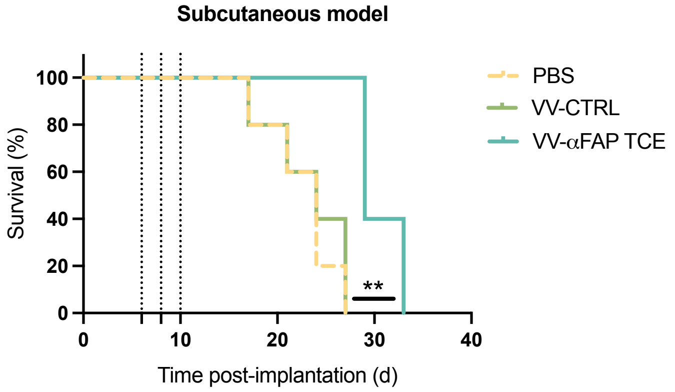
Subcutaneous model
Survival (%)
Time post-implantation (d)
PBS
VV-CTRL
VV-αFAP TCE
**
